# Temporary Seismic Array Installation in the Contursi Terme Hydrothermal System: A Step Toward Geothermal Assessment

**DOI:** 10.3390/s26010016

**Published:** 2025-12-19

**Authors:** Vincenzo Serlenga, Ferdinando Napolitano, Serena Panebianco, Giovannina Mungiello, Tony Alfredo Stabile, Valeria Giampaolo, Massimo Blasone, Marianna Balasco, Angela Perrone, Gregory De Martino, Salvatore Lucente, Luigi Martino, Paolo Capuano, Ortensia Amoroso

**Affiliations:** 1 Consiglio Nazionale delle Ricerche, Istituto di Metodologie per l’Analisi Ambientale, 85050 Tito, Italy; vincenzo.serlenga@cnr.it (V.S.); serena.panebianco@cnr.it (S.P.); valeria.giampaolo@cnr.it (V.G.); marianna.balasco@cnr.it (M.B.); angela.perrone@cnr.it (A.P.); gregory.demartino@cnr.it (G.D.M.); salvatorelucente@cnr.it (S.L.); luigimartino@cnr.it (L.M.); 2 Dipartimento di Fisica “E.R. Caianiello”, Università degli Studi di Salerno, 84084 Fisciano, Italy; fnapolitano@unisa.it (F.N.); g.mungiello@studenti.unisa.it (G.M.); mblasone@unisa.it (M.B.); pcapuano@unisa.it (P.C.); oamoroso@unisa.it (O.A.); 3 Scuola di Ingegneria, Università degli Studi di Potenza, 85100 Potenza, Italy

**Keywords:** seismic array, geothermal energy, local seismic response, earthquake detection, citizen science

## Abstract

How can the interaction between the seismological community and society contribute to the exploitation and usage of renewable energy resources? We try to provide an answer by describing the seismic experiment realized in March–April 2025 in the hydrothermal area close to Contursi Terme municipality (Southern Italy). We deployed a 29-station seismic array thanks to the availability of local citizens, civic administrations, schools, and accommodation facilities, which provided hosting and power for six-component seismological instruments over a one-month period. By computing the Probabilistic Power Spectral Densities (PPSD) and spectrograms, we assessed the noise level and the quality of the dataset. The seismic recordings were also used for studying the local seismic response of the area by the HVSR method and detecting small magnitude (1.4–4.2) local and regional earthquakes. We thus described some solutions to tackle the challenges of a possible geothermal exploitation project in the area: (a) to map the energy resource through a tomography on good-quality ambient-noise data; (b) to manage the seismic risk related to the resource exploitation by installing a proper local seismic network; (c) to increase the acceptance by the population through a citizen-science action for instituting a fruitful alliance between different actors of civil society.

## 1. Introduction

Despite the absence of a unique definition, a seismic array is usually described as a set of sensors deployed at distinct points of a target area. The sensors composing the array, which hopefully should share the same technical features, are positioned with a well-defined spacing and geometry; furthermore, they simultaneously acquire the seismic signals propagating across the subsurface [1]. The main idea behind the use of seismic arrays is that the appropriate processing of the signals recorded at different sites may provide insights into the properties of the seismic wavefield at a certain point, whether inside or in the proximity of the network [1]. It is usually done by studying the correlation of the signal waveforms recorded between adjacent sensors [2]. This seismological monitoring tool, whose full potential was still unknown at the time, came to the fore in the late 1950s: the Cold War concerns enhanced the interaction between the politics and the science, leading to the shared necessity of: (a) increasing the seismological knowledge which, at the time, did not include adequate theories, data and manpower to correctly distinguish nuclear explosions from earthquakes of similar magnitude; (b) developing a tool which could allow for increasing the detectability of nuclear tests with respect to the one already provided by the analysis of seismic data recorded by single three component sensors [3,4,5]. The very fast advancements and efficiency improvements of this seismological monitoring instrument over the years, in addition to the rising availability of cost-effective sensors [6,7,8], led the seismological community to increasingly glimpse its strong potential not only for military applications but for more noble and scientific goals too. In this context of scientific and technological progress, the contribution as the main character of the ambient seismic noise cannot be neglected. The latter, indeed, has experienced an evolution from being a rival to be suppressed for the enhancement of the useful seismic signal to becoming a positive hero in the seismic array novel, especially after the sudden appearance of Shapiro and Campillo [9]. Depending on their aperture (i.e., the largest distance between the stations constituting the array), on their geometry (i.e., irregular, linear, or circular), on the operational period (i.e., temporary or permanent), and on the technical features of the seismic sensors (i.e., broadband or short-period instruments), applications of arrays may be various. These span from large earthquake studies [10], microearthquake monitoring [11,12], earthquake early warning systems [13,14], detection of non-volcanic seismic tremors [15,16], to deep Earth [17] and shallow subsoil investigations and imaging [18]. The latter probes, leveraging recorded seismic waves generated by both earthquakes and ambient-noise natural and anthropic sources, are usually aimed at: (1) gaining insights on the hidden deep geodynamic processes feeding the Earth’s surface (e.g., [19,20] and references therein); (2) investigating the causative drivers of volcanic unrest (e.g., [21]); (3) characterizing the properties of different sites in the framework of local seismic response and microzonation studies (e.g., [22,23] and references therein); (4) assessing the energetic resources’ potential located in the subsurface (e.g., [24,25,26]).

The latter purposes are pivotal at present times and in the near future, especially considering the United Nations’ 2030 AGENDA. Goal 7 first aims at guaranteeing safe, sustainable, and modern energy to the world population and then underlines the key role of energy in sustainable development to counteract Earth’s climate changes as much as possible. Among the main contributors to the climate crisis, there is a consensus on the negative role of greenhouse gas (GHG) emissions, which, together with other factors, are released by the burning of fossil fuels [27]. Hence, there is the necessity, as stated by the AGENDA, to significantly increase the adoption of renewable energy sources by 2030. Furthermore, the European Union aims at reaching climate neutrality by 2030 [28]. In this context, geothermal energy seems to be a kind of silver bullet: clean, safe, renewable, with a minimum level of GHG emissions and constant baseload power. Nevertheless, the still existing downside of this technology lies in problematic aspects such as the high development costs, the long duration of project timelines, and the difficulty in mapping possible hidden reservoirs. Increasing the acceptance and awareness levels of the population is an additional challenge related to geothermal energy exploitation. Finally, the process of circulation of hot and cold fluids strictly linked to the geothermal production may cause, among the others, pressure leakage along active faults [29] or stress variations due to heterogeneous temperature changes [30,31]: therefore, the monitoring of seismicity and, generally speaking, the management of the related seismic risk must constitute a good practice for any planned geothermal energy project [32].

The TOGETHER project “Sustainable geothermal energy for two Southern Italy regions: geophysical resource evaluation and public awareness” funded by European Union—Next Generation EU (PRIN-PNRR 2022) settles in the described societal context, with its dual purpose: (a) to characterize the subsoil in two Southern Italy sites through a multi-messenger approach combining multiple geophysical techniques, in order to provide information relevant to geothermal resource assessment; (b) to establish a fruitful interaction between the subjects involved in the development of a geothermal project, comprising scientists and public institutions, legislators, and, last but not least, citizens.

This study is fully embedded in the goals of TOGETHER. Indeed, it describes an experimental temporary seismic array deployed in the Sele River Valley area between Oliveto Citra, Colliano, and Contursi Terme municipalities, with the latter being one of the two test-bed sites of the project. Contursi Terme, in the Campania region and specifically in the Sele River Valley, is situated in a low heat flux area (30–40 mW/m^2^) [33,34,35]. Here, the geothermal resources located at depths ranging from 500 m to few kilometers define Contursi Terme as a low enthalpy hydrothermal system characterized by water temperature between 21 °C and 47 °C [36], which are mainly adopted for balneotherapy and spa purposes. The Sele Valley is also characterized by the presence of cold and hot degassing vents through which deep CO_2_ and He emerge at the surface [37,38]. From a seismic risk perspective, the target site is very close to the system of faults responsible for the Mw 6.8 November 1980 earthquake that occurred in the sector of Campanian–Lucanian Apennines (Irpinia) [39]. Its closeness to such a hazardous region makes Contursi Terme and Oliveto Citra being in the 2nd zone of the Italian Seismic Classification [40], whereas Colliano is classified in the 1st zone (the highest seismic hazard zone) [40].

The conceptual design of the here presented experiment settles in an intermediate space where the science still meets and engages with people, thus unfolding in these main purposes: (1) to reconstruct a 3-D shear wave velocity tomographic model by means of ambient seismic noise tomography methodology in order to serve the first purpose of TOGETHER project; (2) to foster the interaction between the scientific community and the population in order to increase the people awareness of geothermal energy; (3) to analyze the site response of the area in a seismic risk mitigation perspective; (4) to further gain insights about the capability of earthquake detection at stations also installed in the urban environment, where the anthropogenic noise is higher.

In this manuscript, we first describe the conceptualization of the experiment and the installation of the temporary seismic array in the study area introduced above. Then, we discuss the overall quality of the ambient seismic noise data. Finally, we present the first site amplification results obtained using the HVSR method and report the earthquake recordings acquired during the one-month monitoring period.

## 2. Description of the Experimental Seismic Array

### 2.1. Available Sensing Technologies

**Figure 1:**
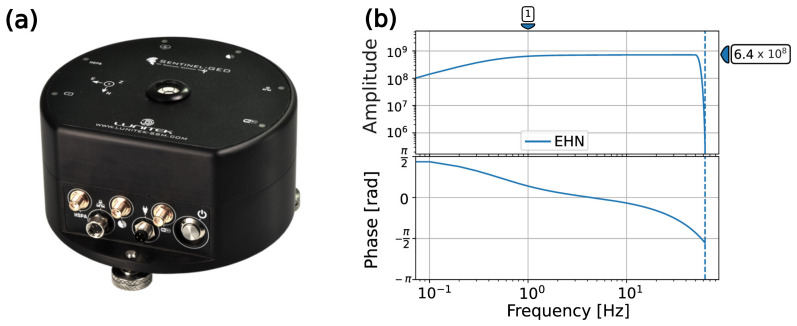
Sentinel GEO MKII (**a**) Picture of the instrument (**b**) Amplitude and phase response curves of the longitudinal component of the velocimetric sensor of Sentinel—GEO MKII, also including the response of the digitizer. The responses are the same for the vertical and transverse components of the sensor, and they are common to all the sensors adopted in the experiment.

In the framework of the ITINERIS project (Italian Integrated Environmental Research Infrastructures System), funded by the European Union—Next Generation EU Program, the Institute of Methodologies for Environmental Analysis of the Italian National Research Council (CNR–IMAA) acquired an equipment of 35 Sentinel GEO MKII by the Lunitek company (Sarzana, Italy). The Sentinel GEO MKII is a high-quality accelerograph with an embedded triaxial velocimeter, and it was mainly conceived for both structural monitoring and seismological studies with budget-sensitive constraints. These relatively low-cost sensors contain a triaxial MEMS accelerometer with a dynamic range of 85 dB and three 4.5 Hz geophones. Each one is characterized by a sensitivity of 700 V/m/s and is electronically extended down to 2 s by means of a built-in digitizer (Figure 1). The upper frequency bound of the geophones is set to 100 Hz but is still subject to: (1) the Nyquist frequency, in turn depending on the sampling rate; (2) the presence of an anti-alias Finite Impulse Response (FIR) filter. The sensor has a 32 Gb memory card, and it is equipped with its own internal battery (LiPo), which can guarantee autonomy for up to 4 h. Therefore, to allow for continuous monitoring and long-term data acquisition, Sentinel GEO MKII is powered by an external DC source (9–36 VdC). The synchronous acquisition by the whole sensors’ equipment is ensured by the presence of a GNSS receiver, which provides absolute UTC. The sensor could be accessed both via Wi-Fi–LAN connection and via a remote connection through an internal 4G modem. Nevertheless, at the time of the experiment, the latter was not yet internally installed and, therefore, only the former (local) connection modality was available.

### 2.2. Coherency of Green’s Function in Neighborhood Areas

In the preliminary phase preceding the design and deployment of the seismic array, we posed a fundamental question: are the ambient seismic noise conditions of the target area favourable for a subsurface investigation based on seismic array techniques, and in particular on ambient seismic noise tomography?

**Figure 2:**
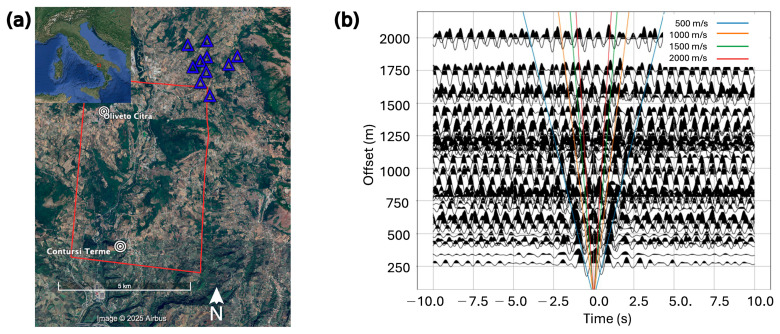
(**a**) Position of the array “08” of the DETECT experiment (ten seismic stations represented by blue triangles) with respect to the target area of this study (red box). Study area as shown in Google Earth imagery (base imagery © Google, © Maxar Technologies, 2025) [48]. The geographical positions of Contursi Terme and Oliveto Citra municipalities are reported. (**b**) Cross-correlation functions of the vertical components (ZZ), stacked over 12 days, filtered in the frequency range 1.0 Hz–2.0 Hz, and organized as a function of the interstation distance.

Both experimental and numerical studies have shown that the preferential condition for retrieving a coherent Green’s function from the cross-correlation of seismic noise recorded by pairs of stations is the presence of an isotropic and stochastic distribution of noise sources surrounding the array [9,41]. In turn, the computation of such coherent Green’s functions represents the fundamental of the ambient seismic noise tomography methodology [42,43]. In this regard, in a regional tomographic study of the Campania–Lucania sector of the Apennines (Southern Italy), Vassallo et al. [44] already made use of ambient seismic noise recorded by, among others, seismic stations COL3 and CDRU, respectively, belonging to ISNet [45] and Rete Sismica Nazionale (RSN) seismic networks. The ray paths connecting these two stations cross an area close to the target of our experiment, providing us with a first positive, but not exhaustive, answer about the favourable noise conditions in the area. To completely address it, we decided to analyze a small portion of the seismic data collected by the DETECT experiment [46] carried out in the period August 2021–August 2022 in the Campania–Lucania sector of the Apennines. This experiment consisted of deploying a constellation of 20 seismic arrays along the fault system responsible for the above cited 1980 Irpinia earthquake. Each seismic array was composed of 10 seismic stations, and in particular of one broadband sensor (Trillium − Compact/g = 750, Nanometrics, Ottawa, ON, Canada), one 1 s short-period sensor (Mark L4–3D, Sercel, Carquefou, France), and eight 4.5 Hz geophones. To our purposes, we analyzed the continuous data stream recorded by the array “08” located about 5 km north-east of Contursi Terme (Figure 2a), with the aim of assessing the coherency of the Green’s function estimated from the cross-correlation of the data recorded by all the pairs of stations. Effectively, we operated on about three weeks of continuous ambient-noise data simultaneously recorded with a sampling frequency of 200 Hz by the ten seismic stations composing the array “08”. Our analysis was limited to the vertical component of the collected seismic signals. We pre-processed the daily seismic data of each seismic station by mean and trend removal and instrument correction, obviously taking into account the differences between the sensor responses. Finally, the seismic data were band-pass filtered in 0.5–1 Hz and 1–2 Hz frequency ranges. The processed seismic data were then split into 6-h-long time windows starting from midnight each day. After that, the cross-correlation (CC) between each pair of stations was computed. Finally, the cross-correlation functions were stacked over three different time intervals: 3 days, 12 days, and 16 days. By visual inspection, we observed that the cross-correlation functions computed by stacking 12 and 16 days were very similar, allowing us to assert that after 12 days the CC stack becomes stable, as also retrieved by Vassallo et al. [47] in a similar experiment carried out in an urban environment.

The vertical, stacked cross-correlation functions computed for all the possible station pairs of the array are shown in Figure 2b. The CC functions, obtained on seismic signals filtered in the frequency range 1.0–2.0 Hz, are plotted as a function of the interstation distance reported on the *y*-axis. We observe an energetic seismic phase in the cross-correlograms, both in the causal and in the a-causal parts. This seismic phase is very clear up to an interstation distance of about 750 m, and the theoretical dromocrones superimposed on the CCs allow an approximate estimation of its propagation velocity between 500 m/s and 1000 m/s. At higher distances, this travelling symmetric phase seems to be characterized by a low signal-to-noise ratio, and it is no longer evident. It is worth remembering that this preliminary investigation has been carried out by analyzing ambient seismic noise data mainly collected by 4.5 Hz geophones, which are not sensitive to the lower frequency range, where usually the Green‘s function coherency is most evident.

### 2.3. Design and Deployment of the Seismic Array

The approximate location of installation sites of the array’s stations was chosen in laboratory in order to fulfil the following criteria: (1) to cover the positions of some of the geothermal sources and springs known in the area [36,49], by guaranteeing also crossing paths for retrieving lateral velocity anomalies through tomography; (2) to span a wide range of station spacings which is a requested condition for any array experiment aimed at the subsurface characterization: in that way both short and long wavelength may be investigated; (3) to gain insights on areas characterized by different surface geological conditions and, therefore, by different seismic responses; (4) to ensure an array geometry as circular as possible, to take into account the inevitable anisotropic distribution of noise sources and to reduce the azimuthal dependence of the array response function. Furthermore, we sought to design an array with the maximum distance between the seismic stations (aperture of the array) close to 10 km, to reach a higher investigation depth in our future tomographic studies. Finally, we decided to schedule a continuous acquisition of ambient seismic noise lasting about one month, given the proven efficiency of this duration in providing 3-D images of the subsoil through ambient seismic noise tomography (e.g., [24,25]).

The experiment was carried out in the late winter and early spring of 2025. It started with a scouting activity aimed at selecting the best sites for the installation of the sensors, first based on the laboratory plan described above. Due to the reduced battery autonomy of the available sensors and to their necessity to be connected to the power grid for the planned one month acquisition, the scouting activity mainly consisted of the interaction with local people, schools, accommodation facilities, civic administrations: their availability to host and to power a seismic sensor for the planned period of acquisition was indeed the first necessary (but not sufficient) condition for the success of the array experiment. In that way, we were able to pursue both the scientific and the dissemination purposes of the experiment and of the TOGETHER project, in a wider perspective. Both private sites and public sites were chosen: among the former, there were uninhabited country houses, cellars, huts, and farm stalls. We also collected the availabilities from restaurants, B&B owners, and a spa hotel. Finally, the interaction with civic administrations and schools allows the identification and the availability of two spaces, which are a public school and a municipal kindergarten, in Contursi Terme and Oliveto Citra, respectively.

**Figure 3:**
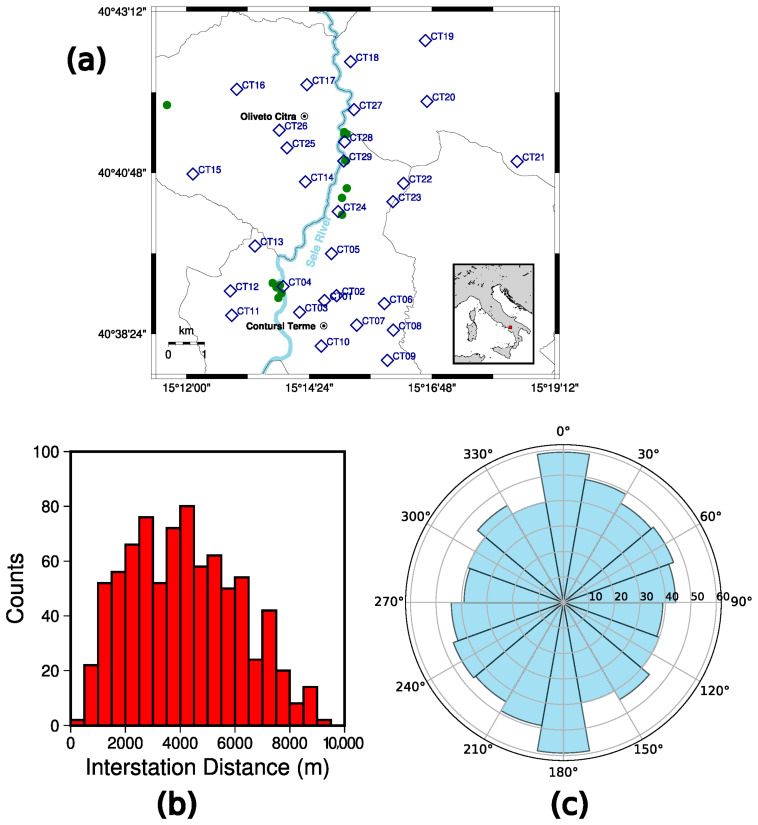
Seismic array installed in the target area (**a**) Map of the installed seismic stations: the blue diamonds represent the seismic stations with the respective station name as reported in Table 1, whereas the green points identify the springs as retrieved from [36]. The Sele River is also reported on the map by the light cyan path. (**b**) Histogram of the interstation distances between all the station pairs of the experiment. (**c**) Histogram of the azimuths of the imaginary paths connecting all the pairs of stations. The computation of the azimuths has been performed through the WGS84 ellipsoidal projection by the QGIS software (version 3.30.3).

The installation of 29 seismic stations took place in the first week of March 2025, from the 3rd to the 7th of March (Table 1, Figure 3a). The final deployment of the sensors, which was necessarily adapted to the logistical constraints explained above, provided an interstation distance ranging from 370 m to 9.2 km, with an average value of about 4 km (Figure 3b). Furthermore, the imaginary paths connecting all the possible station pairs (406) span the whole azimuthal range, with a predominance of the azimuths between 330° and 30° (Figure 3c). Indeed, along the Sele River and the municipal roads connecting the southern and northern parts of the target area, we collected a higher availability from local people with respect to the most isolated fields and countryside located east and west of the Sele River. The latter, furthermore, constitutes a meaningful and directive source of noise in the context of our experiment; therefore, it is very useful to catch as much as possible the contribution of the river to the ambient seismic noise acquisition.

The sensors were installed, based on the specific site conditions, both at the basement level and in free-field conditions. In the latter situation, the sensor was still protected from rain by installing it below canopies. All the sensors were installed orienting their longitudinal axis toward the geographic north by adopting a geologist’s compass. The acquisition started by setting a sampling frequency of 125 Hz, corresponding to a Nyquist frequency of 62.5 Hz. The unavailability of the 4G modem installed inside the sensors when we deployed the array did not allow us to remotely connect to the seismic sensors during the acquisition period to check the proper functioning of the stations and possible recording anomalies.

The seismic array was undeployed on the 4th and the 8th of April 2025, thus providing us with about one month of simultaneous acquisition by the installed Sentinel GEO MKII sensors.

## 3. Results

### 3.1. Data Availability and Overall Quality of the Dataset

**Figure 4:**
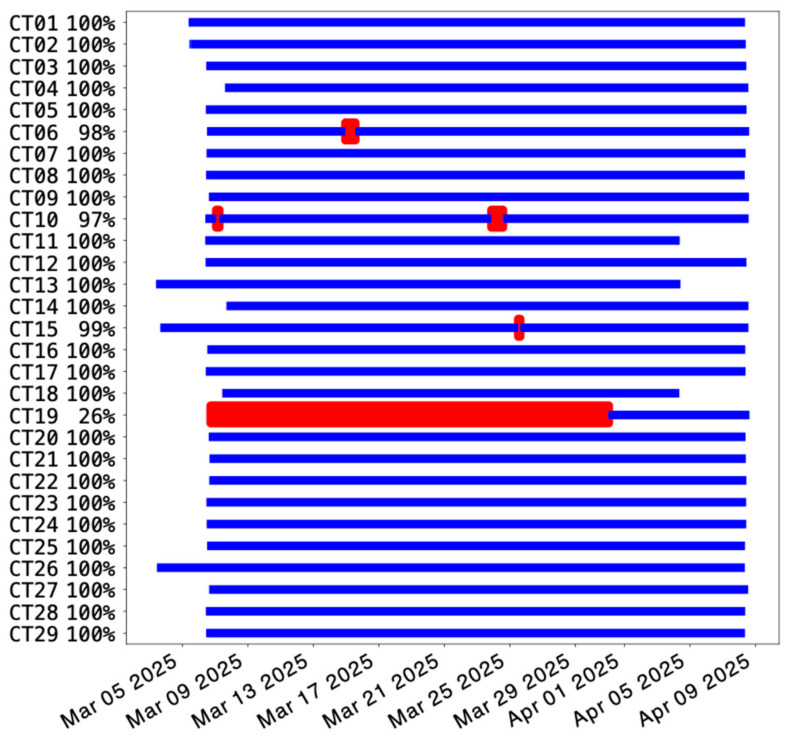
Availability of seismic data over the acquisition period; only the velocimetric vertical components of the sensors have been used to produce this image, given the equivalent availability at the longitudinal and transverse components. The red vertical sectors represent the discontinuity periods in the data acquisition. On the left, for each station, the truncated percentage of available data is reported.

The installed temporary seismic array recorded data with an optimal continuity, as shown in Figure 4, with a very limited data loss; indeed, the four-hour autonomy battery of the installed Sentinel GEO MKII allowed the system to cope with occasional power failures during this period. A total of 25 stations out of 29 have 100% recorded data over the month of acquisition. A significant problem occurred to stations CT19, located at the north-easternmost limit of the array, which deleted the first 20 days of continuous data due to an issue related to the configuration of acquisition.

**Figure 5:**
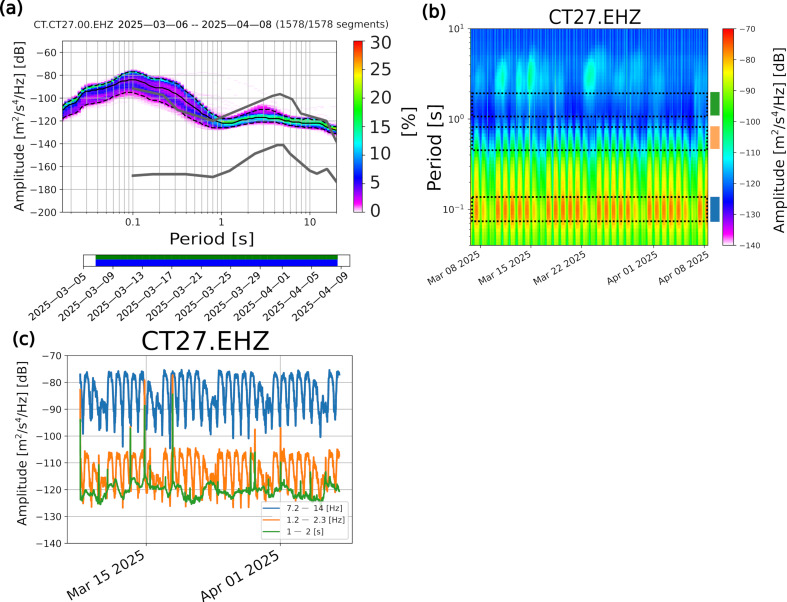
Spectral analyses on the vertical component of CT27 station recordings. (**a**) Probabilistic power spectral density (PPSD): the grey solid lines represent the NLNM and NHNM curves. The black solid line identifies the 50th percentile of the probabilistic distribution; the lower and upper dashed solid lines represent the 5th and 95th percentile curves, respectively. The lower green and blue bars indicate the available data and the single PSD measurements that go into the histogram, respectively. The color palette on the right indicates the probability (in percent) that a certain level of noise is present. (**b**) Spectrogram of the CT27 station over the whole period of acquisition. The black dashed rectangles identify the period ranges over which the PSD time series has been computed. The blue, orange, and green rectangles at the right of the spectrogram define the color code adopted for each frequency range and used for displaying the PSD time series reported in panel (**c**). (**c**) PSD time series over the whole period of acquisition. The curves identified by different colors refer to the frequency ranges highlighted in panel (**b**)**.**

**Figure 6:**
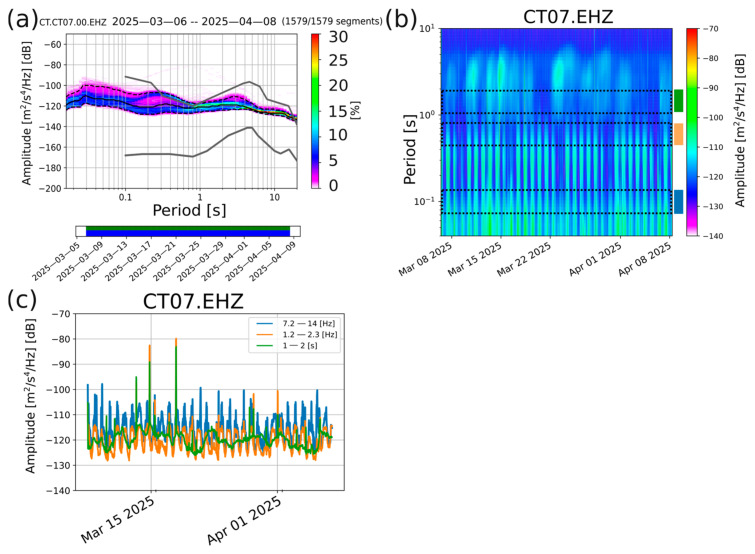
Spectral analyses on the vertical component of CT07 station recordings. (**a**) Probabilistic power spectral density (PPSD): the grey solid lines represent the NLNM and NHNM curves. The black solid line identifies the 50th percentile of the probabilistic distribution; the lower and upper dashed solid lines represent the 5th and 95th percentile curves, respectively. The lower green and blue bars indicate the available data and the single PSD measurements that go into the histogram, respectively. The color palette on the right indicates the probability (in percent) that a certain level of noise is present. (**b**) Spectrogram of the CT07 station over the whole period of acquisition. The black dashed rectangles identify the period ranges over which the PSD time series has been computed. The blue, orange, and green rectangles at the right of the spectrogram define the color code adopted for each frequency range and used for displaying the PSD time series reported in panel (**c**). (**c**) PSD time series over the whole period of acquisition. The curves identified by different colors refer to the frequency ranges highlighted in panel (**b**).

To describe the level of background seismic noise recorded by the 29 stations composing the seismic array, we first corrected the continuous data stream for the instrument response. Then, we computed the Probabilistic Power Spectral Densities (PPDS) of the seismic recordings by the three components in the whole period of acquisition. To our purposes, we adopted the algorithm developed by McNamara and Buland [50] implemented in the open-source PPSD signal processing routine contained in the ObsPy package (a Python framework for processing seismological data [51]). The PPSD algorithm analyses the continuous data stream, splitting it into one-hour-long time windows, with a 50% of overlap between the different time segments for reducing the Power Spectral Density (PSD) variance. The computed PPSD were then compared with the New-High Noise Model (NHNM) and New-Low Noise Model (NLNM) curves obtained by Peterson [52]. In addition, to explore the changes in time of the spectral power of the seismic noise recordings, we computed the spectrograms and the time series of the PSD in three selected period (frequency) ranges by adopting the same cited algorithm [50]. We here show two examples of PPSD, spectrograms, and PSD time series, the latter computed in three distinct frequency ranges (0.5–1 Hz green curve, 1.2–2.3 Hz orange curve, 7.2–14 Hz blue curve in Figure 5 and Figure 6); in particular, the chosen cases refer to the analyses carried out on the vertical-component ambient seismic noise recordings at two distinct sites, namely CT27 and CT07, representing two opposite end-members: a very noisy and a very quiet place, respectively. The former was both close to a private bus station and to a congested highway, whereas the latter was in the middle of the country, east of the Contursi Terme municipality. The median PPSD curve (solid black line) of the noisy site (CT27) (Figure 5a) exceeds the NHNM curve (upper solid grey line) at frequencies greater than 1 Hz (periods lower than 1 s) by a value of approximately 10 dB. It highlights the strong contribution of anthropic noise in the vicinity of the recording site. On the other hand, the microseismic contribution, related to periods greater than 1 s, lies between the NLNM and NHNM curves. The general trend of the PPSD median curve is compatible with the Peterson noise models up to periods equal to about 3 s, and this observation agrees with the characteristics of the seismometer and its amplitude response curve; nevertheless, the 0.5 Hz cut-off frequency of the Sentinel GEO MKII hides the secondary microseismic peak, which is the strongest noise peak, usually located between 4 s and 6 s [53]. The analysis of the spectrogram and of the PSD time evolution allows highlighting some interesting features (Figure 5b,c): the day and night noise amplitude variations (on average equal to 20 dB) in the 7–14 Hz frequency range (blue curve of Figure 5c) are very clear. Indeed, this interval is the one in which the anthropic contribution is very relevant, and it distances itself from the natural sources’ effect, mainly concentrated at frequencies lower than 1 Hz. In addition, it is remarkable the strong amplitude reduction (about 10 dB) starting from the mid of the Saturday of each recording week (for sake of clarity, we remind you that most of the sensors were installed the 6th of March (Table 1), which was a Thursday): indeed, the private bus station mainly serves the transportation of students among the school institutes of the municipalities settled around the investigated area (i.e., Contursi Terme, Oliveto Citra and Colliano); we still note the proximity of this site to a highway road. The same highlighted features are clear in the frequency range 1.2–2.3 Hz, represented by the PSD time evolution orange curve (Figure 5c): the difference between day and night amplitudes is lower (on average approximately equal to 15 dB). Nevertheless, the weekend drop is compatible with that observed in the highest frequency range.

The quieter site (CT07), on the other hand, shows a PPSD curve (Figure 6a) which is always within the limits defined by the Peterson curves, being characterized by lower levels of spectral power. The PPSD trend still resembles Peterson’s in the low frequency range, up to a period value of 3 s, due to the cut-off frequency of the Sentinel GEO MKII of 0.5 Hz. This feature is very evident, especially by looking at the 95th percentile PPSD curve of Figure 6a (upper dashed black line).

Overall, most of the PPSD computed at all the stations and seismic components (Figure S1) show the 50th percentile curve very close to the New-High Noise Model by Peterson; this observation highlights the high level of background noise in the area where the experiment has been carried out. In detail, the seismic stations CT11, CT12, CT17, CT18, CT27, CT28, and CT29 exhibit the average noise level at the lowest periods (0.2–1 s) just above the NHNM curve; the remaining nodes of the seismic array are characterized by average noise levels below the New-High Noise Model.

Both the spectrogram and the time series of the PSD (Figure 6b,c) show the variations between night and day. Nevertheless, these oscillations are lower than the CT27 ones (about 15 dB in the 7–14 Hz range). It is noteworthy that the amplitude overlap between the 7–14 Hz and 1.2–2.3 Hz PSD time series is greater than that observed at the noisier site. It confirms the attenuation of the anthropic noise due to the distance of this site from the inhabited centers, industrial activities, and the most congested roads. The green curve, describing the time evolution of the PSD in the lowest analyzed frequency range, exhibits a general evolution compatible with the noisier site; the peak positions, as well, are completely comparable. This consistency, confirmed also by the PSD time series of the rest of the stations (Figure S2), is a further footprint of the contribution of ubiquitous natural sources in the target area. Furthermore, the peak time positions (especially those that occurred between the 14th and the 15th and the 17th and the 18th of March) are very consistent with the windiest days in the target area over the whole month of acquisition (Figure S3). On the other hand, the days characterized by lower amplitude levels in the 1–2 s PSD times series (e.g., 20 March–27 March) coincide with an average low wind speed. The respective meteorological data have been retrieved from [54].

#### 3.1.1. Stations’ Issues

##### Misorientation of Sensors

**Figure 7:**
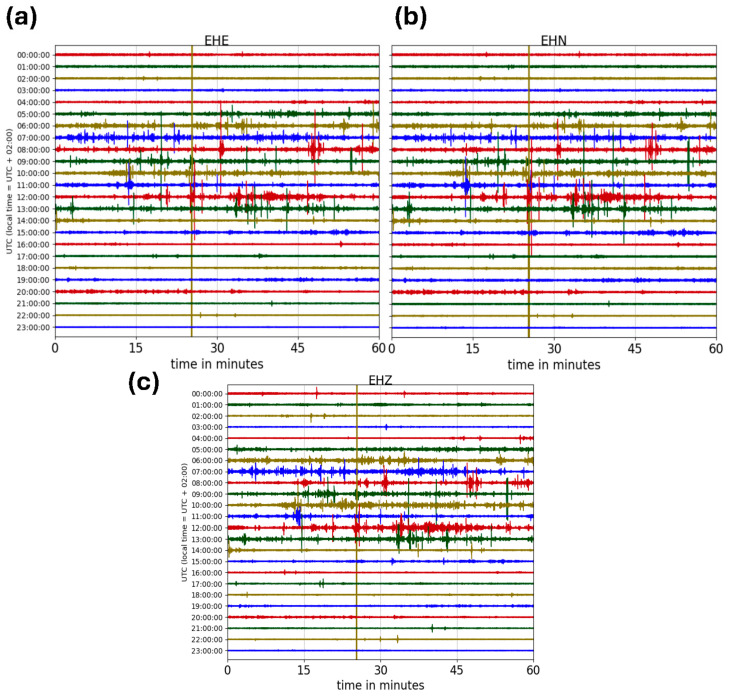
(**a**) Daily helicorder of 30 March (Julian day 89) of the transverse component of the station CT13. (**b**) Daily helicorder of 30 March 2025 (Julian day 89) of the longitudinal component of the station CT13. (**c**) Daily helicorder of 30 March (Julian day 89) of the vertical component of the station CT13.

Despite the good level of noise recorded by the seismic sensors installed in the investigation sites, at the moment of the demobilization of the array, we noticed a misorientation of four stations out of 29, and in detail, CT06, CT10, CT13, and CT15. Since we did not know if the rotation occurred in a single or multiple instances, we did not accurately measure the final orientation errors, but we were just able to notice rotations close to 180° for CT06 and CT15, close to 90° for CT13, and lower than 20° for CT10. The CT13 station was installed in an empty room of a bed and breakfast, which was not visited by any guest during the experiment, and only rarely by the owner, but in separate rooms. Nevertheless, he provided us with an approximate date (between 28 and 30 March 2025) at which the weather conditions caused the accidental fall of the GPS antenna connected to the sensor and externally placed for the correct time synchronization (i.e., on the roof tile above the room window). The relocation of the GPS antenna by the owner (still connected to the sensor) caused the slight movement and the consequent rotation of the seismometer. By looking at the daily helicorders of the three components of the CT13 station (Figure 7), we noticed an abrupt transient (at about 10:25 UTC on 30 March), different from the ones that we observed on previous and next seismic recordings.

**Figure 8:**
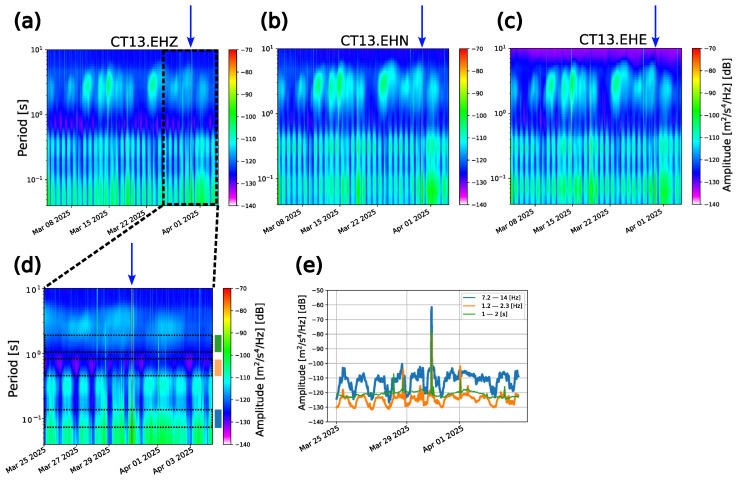
(**a**) Spectrogram of the whole data stream of the vertical component of the CT13 station. The dashed black rectangle delimits the time interval used to compute the spectrogram represented in panel (**d**). (**b**) Spectrogram of the whole data stream of the longitudinal component of the CT13 station. (**c**) Spectrogram of the whole data stream of the transverse component of the CT13 station. The blue arrow on the top of the figure indicates the date corresponding to the abrupt transient observed in the time domain. (**d**) Spectrogram of the selected time interval (25 March–4 April 2025) recordings of the vertical component of the CT13 station. The blue arrow (as in (**a**–**c**)) indicates the temporal position of the spectral footprint related to the abrupt transient identified in the time domain signals on 30 March. The black dashed rectangles indicate the period ranges over which the PSD time series has been computed. The colored blue, orange, and green rectangles at the right of the spectrogram define the color code adopted for each frequency range and used for displaying the PSD time series reported in panel (**c**). (**e**) Vertical-component PSD time series in three distinct frequency ranges: a clear peak in the spectral power is observable on 30 March.

A simultaneous anomalous behavior is also observed in the related spectrograms (on the three components) and in the temporal evolution of PSD, showing an anomalous spectral power peak at all the frequencies (greater than −80 dB) (Figure 8).

The discussed analyses and the joint signal anomalies both in the time and frequency domain, therefore, provided us with some hints to identify, also on the other stations, the moment(s) at which the sensor was unfortunately rotated. On these grounds, we looked at both the daily helicorders of the affected sensors and their spectrograms. For the CT15 station, the same joint signatures already discussed for CT13 were noticed at about 8:30 UTC on the 24th of March. For station CT10, the observation of the helicorder pointed out only one abrupt time domain transient comparable with the ones previously introduced, which occurred 30 min after the installation. Since the computational algorithm [50] uses time windows of 1 h, both spectrograms and PSD do not show a clear variation of the amplitude at the very beginning of the acquisition: indeed, only in the first analyzed window do we observe very high values of the spectral power across all the frequency ranges, and then they come back to more common levels. The latter station affected by such kind of misorientations was CT06: the contextual analysis of both time and spectral domain signals allowed us to identify at least five distinct critical instants at which both the velocity amplitudes and the spectral powers (at all the analyzed frequency ranges) noticeably increase, with respect to the usual night and day oscillations.

##### Malfunction of Sensors’ Component

**Figure 9:**
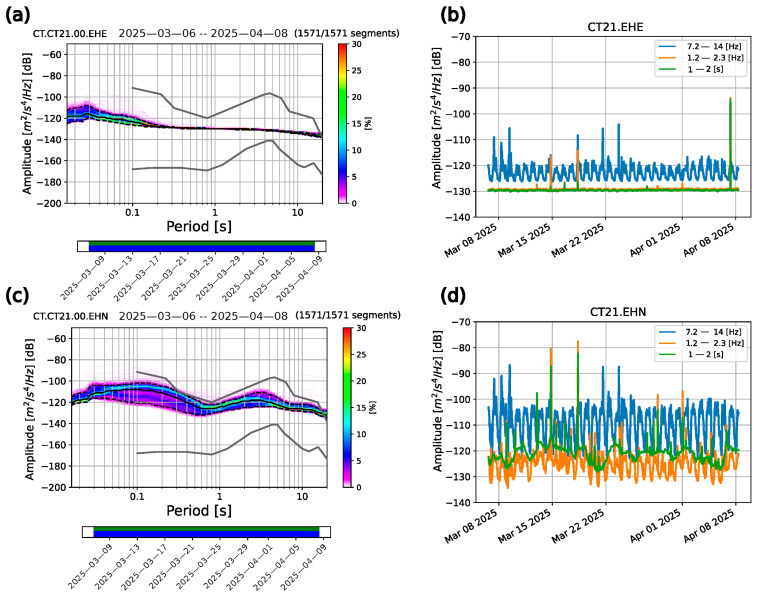
(**a**) PPSD of the continuous data stream recorded by the transverse component of the CT21 station. (**b**) PSD time series computed on the transverse component of the CT21 station in the three selected frequency ranges. (**c**) PPSD of the continuous data stream recorded by the longitudinal component of the CT21 station. (**d**) PSD time series computed on the longitudinal component of the CT21 station in the three selected frequency ranges.

By looking at both the time and frequency domain signals, we noticed an additional problem that affected three stations of the seismic array, namely CT06, CT22, and CT21, consisting of the malfunction of the longitudinal (for the CT06 station) and transverse (CT22 and CT21 stations) components. For the sake of clarity, we here show only an example of the spectral footprints of the damaged sensor transverse component of the CT21 station (Figure 9). In detail, we show the comparison of the PPSD and the PSD time series computed on two distinct components. Both the low spectral amplitudes in the PPSD and the flat distribution of the power spectra computed over the entire month of recordings for the transverse component are clearly evident (Figure 9c). The anomaly is even more evident when comparing this PPSD with the longitudinal one. Furthermore, the variations of spectral amplitudes between night and day, conversely to what already discussed for all the other stations, are observable only in the highest frequency range (7.0–14 Hz, blue curve of Figure 9b), but with a lower level of amplitude: indeed, a difference of more than 20 dB is retrieved between the transverse and longitudinal component peaks (Figure 9b–d). The PSD time series in the 1.2–2.3 Hz range is, instead, almost completely flat, with very rare peaks and no daily oscillations.

##### 1 Hz Peak on the Vertical and Longitudinal Components

The detailed observation of the velocity spectra at all the components (horizontal and vertical) and at all seismic stations composing the seismic array drew attention to an anomalous spectral signature at the frequency of 1 Hz: a spurious peak at this frequency has been observed on the longitudinal and vertical-component spectra of the seismic data recorded by every node of the array. Due to the different outcropping lithologies and stratigraphic conditions of the site where the stations were installed (further details in Section 3.2), we discarded the possible option of a stratigraphic effect on the observed spectra. Furthermore, the discussion with Lunitek, manufacturer of the Sentinel GEO MKII sensors, indicated that the 1 Hz spectral peak arose from an instrumental malfunction, more common when the sensors run on battery power, which is not the case in our experiment. The further analyses carried out by us to gain insights on this issue, as thoroughly described in Figure S4 in the Supplementary Materials, allowed us to demonstrate that this anomaly is cancelled out by a proper smoothing procedure through the Konno–Ohmachi algorithm.

### 3.2. Horizontal-to-Vertical Spectral Ratios (HVSR)

The seismic ambient-noise recorded during the experiment has been used to evaluate site effects at the station’s location through the horizontal-to-vertical spectral ratio (HVSR) methodology. The basic assumption of this technique [55,56,57] is that the vertical component is free of any influence related to soil conditions at the recording site. Therefore, this method has proven very effective to estimate the site response in the case of simple layered structures, making the result less obvious in the case of complex structures. In addition, its low cost and easy applicability make this technique extensively used for microzonation purposes [58]. The HVSR technique computes the ratio between the horizontal and the vertical spectral components [59], and whether any site effects appear at a certain frequency, it is represented by a peak at a frequency (f_0_) called the resonance frequency of the site. This frequency is also related, in the simplified assumption of a horizontal layered structure, to the S-wave velocity (V_S_) of the resonant soil and to its thickness (H) by f_0_ = V_S_/4H [60].

**Figure 10:**
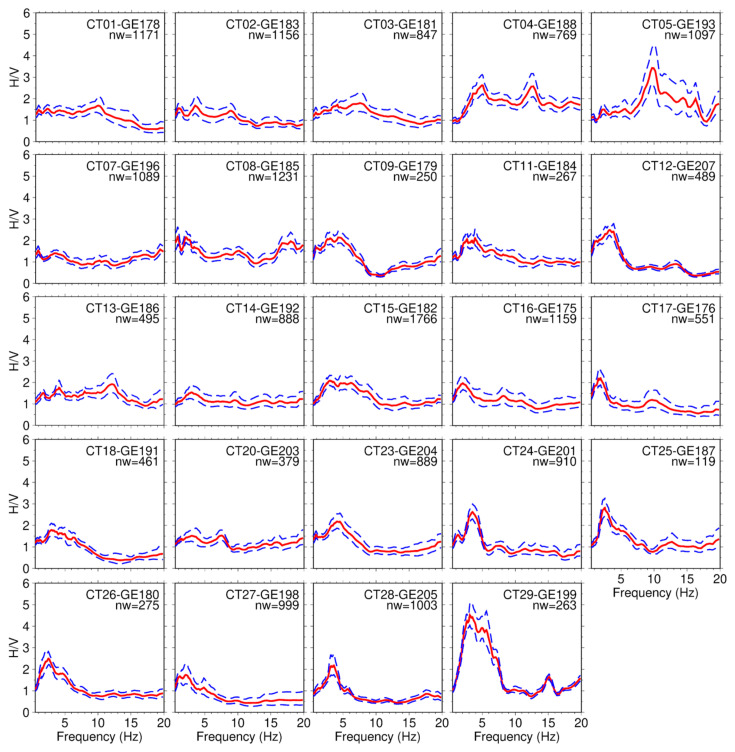
HVSR curves computed on the data recorded in the first week of the experiment by the seismic stations composing the array installed in the target area. Only the results related to stations that did not present any recording problems have been shown. On the top right of each graph station name, the respective sensor ID, and the number of time windows (nw) adopted for HVSR computation are reported; the solid red curve represents the average HVSR curve, whereas the blue dashed lines identify the standard deviations.

To perform the HVSR analysis, we used the Geopsy tool [61]. HVSR was computed using 120 s time windows with an anti-triggering algorithm (Short time average/long time average—STA/LTA) to avoid large transient signals and tapered with a 5% Tukey window type. The spectra have been smoothed with the Konno–Ohmachi procedure (logarithmic width = 5%). The large amount of data (one-month recordings) allowed us to select the most suitable recordings to bring out a high-quality analysis from the data. Since some of the stations could have been affected by weather conditions, we preferred to perform our analysis using data from the first week of acquisition, when there were sunny and not windy days. Furthermore, the analysis described in Section 3.1.1 guarantees the right orientation of the sensors in that period, except for stations CT10 and CT06. We also excluded from our analyses stations CT22 and CT21, whose transverse components were malfunctioning. We selected night-time hours (9 p.m.–7 a.m.) to reduce the amount of anthropogenic noise, for a period between 4 and 5 days at each seismic station. After applying the anti-triggering algorithm and manually removing some remaining anomalous transients by visual inspection, we ended up with a total number of HVSR windows varying from a minimum of 119 windows to a maximum of 1766. The mean HVSR value and the computed standard deviation for each seismic site are shown in Figure 10.

**Figure 11:**
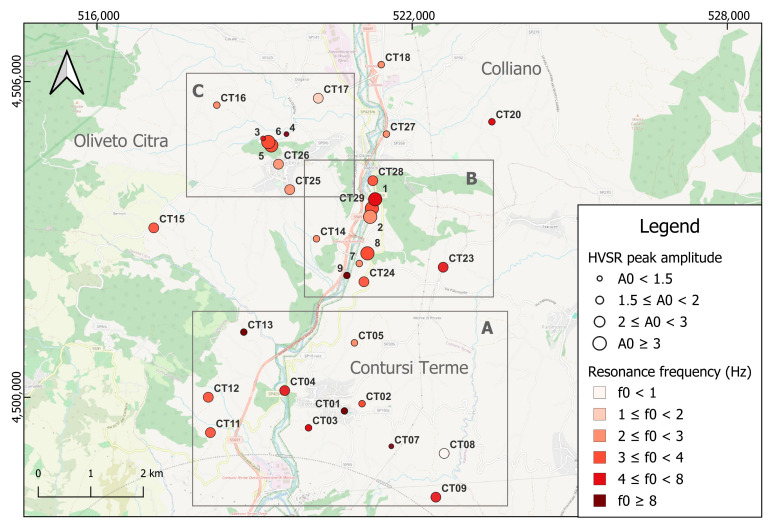
Spatial distribution of the resonance frequencies and amplitudes of HVSR peaks in the target area. The A, B, and C boxes group three different areas, characterized by distinct resonance frequency and peak amplitude features. The geographical coordinates are expressed in terms of UTM Easting and Northing (Longitude Zone 33, Latitude Zone T).

The HVSR curves exhibit significant variability across the study area, which correlates well with the local geology. To enhance the clarity and interpretability of the results, we present, alongside the HVSR curves for all stations, a summary map showing the peak frequency and corresponding amplitude, as shown in Figure 11. It is well known that the HVSR technique tends to underestimate the actual amplification factor due to the theoretical assumptions underlying the method [62,63]. Nevertheless, in this analysis, it serves as a useful proxy for distinguishing areas of higher amplification than others and relating these differences to the local geology.

Summarizing the results, 16 out of the 24 sites (five stations characterized by the above-introduced recording issues have been discarded from the final analyses) do not exhibit significant amplification effects, displaying amplitudes across the entire frequency range (0.5–20 Hz) that remain below or around a value of 2. Three out of these 16 stations, namely CT07, CT10, and CT20, show amplitudes below 1.5 and an average HVSR curve that is flat at all frequencies, clearly indicating a complete absence of amplification at these sites. The other 12 stations exhibit an amplification value between 1.5 and 2, suggesting a low, but not negligible, impedance contrast between the layers involved in the uppermost meters of the subsurface. Finally, the remaining 8 sites exhibit peak amplitudes greater than 2, reaching a maximum value of 4.5 around 3 Hz at station CT29. The resonance frequencies of these peaks are quite comparable, with just the CT05 station exhibiting a high-frequency peak around 10 Hz, whereas the others show peaks at lower frequencies (between 2 and 5 Hz).

**Figure 12:**
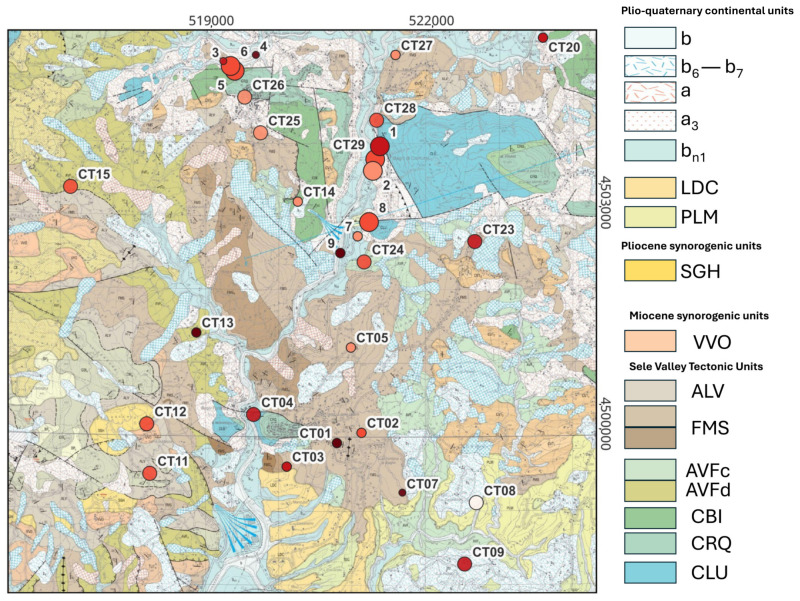
Regional geological map (1:25,000), sheet 468 “Eboli”, South-East section [64] (by permission and for use by ISPRA—Italian Geological Survey Department), with the superposition of peak amplitudes and fundamental frequency as retrieved by the HVSR analyses at the different stations of the seismic array and already reported in Figure 10. b: alluvial deposit; b_6_–b_7_: eluvial product–colluvial deposit; a: slope deposit; a_3_: talus material; b_n1_: terraced deposit; LDC: S. Licandro system, mainly composed of gravel and sands; PLM: PLM: Palomonte syntheme, mainly composed of conglomerates; SGH: Saginara clays and silty clays; VVO: Vallone Vonghia conglomerates and sandstones; ALV: Superior varicoloured clays; FMS: Formazione di Monte Sant’Angelo, mainly composed of limestones; AVF_c_–AVF_d_: Inferior varicoloured clays; CBI: Bio-litoclastic limestones; CRQ: Limestones; CLU: Limestones and dolomitic limestones. The geographical coordinates are expressed in terms of UTM Easting and Northing (Longitude Zone 33, Latitude Zone T). The geological map is publicly accessible at https://sit2.regione.campania.it/content/geologia-geotematismi-itinerari-geologico-ambientali (Foglio 468_Eboli.zip, last accessed on 15 October 2025) [64].

A comparison with the national geological map (Figure 12) reveals that the HVSR curves from the stations exhibiting peak amplitudes < 2 are mainly located on limestones (FMS, CBI, CRQ, CLU units in Figure 12) or Flysch formations (SGH, VVO units in Figure 12) in the whole study area. Conversely, the stations showing higher HVSR peak amplitudes > 2 are situated in areas characterized by the presence of clays, terraced alluvial deposits, and eluvial/colluvial materials (b, b_6_–b_7_, b_n1_, SGH, ALV, AVF units in Figure 12). Particular attention should be given to the central area of the map, corresponding to the Bagni di Contursi area, where the main thermal facilities are located. These are naturally situated near the principal surface manifestations, which exhibit elevated temperatures (up to approximately 47 °C). In this very area, the most prominent HVSR peaks are observed (CT28, CT29), confirmed by additional single-station measurements conducted at different times and not described in this study. These peaks are associated with terraced alluvial deposits (b and b_n1_ units) that thicken progressively from east to west, towards the Sele River. 

Where possible, the results of these analyses have been corroborated by independent geophysical investigations, such as MASW measurements carried out within the framework of the first-level seismic microzonation of the municipality of Contursi Terme, as well as single-station ambient-noise measurements and electrical resistivity tomography (ERT) surveys conducted as part of the TOGETHER project.

### 3.3. Earthquake Recordings

During the one-month deployment of the temporary seismic array in the Contursi Terme area, the visual inspection of the daily helicorders of seismic stations allowed the detection of several earthquakes: we identified 11 local earthquakes, located within a maximum distance of about 50 km from the center of the array, and ranging in magnitude between 1.4 and 4.2. In addition to them, we also recorded seismic events which occurred still in Southern Italy, but at greater distances: in the Campi Flegrei caldera, along the Adriatic Coast (Costa Garganica), and in the Calabria region, approximately 100 km, 150 km, and up to 200 km away, respectively. A total of 35 earthquakes were recorded. For the sake of simplicity, only the recorded earthquakes with epicentral distances less than 50 km from the array are listed in Table 2.

**Figure 13:**
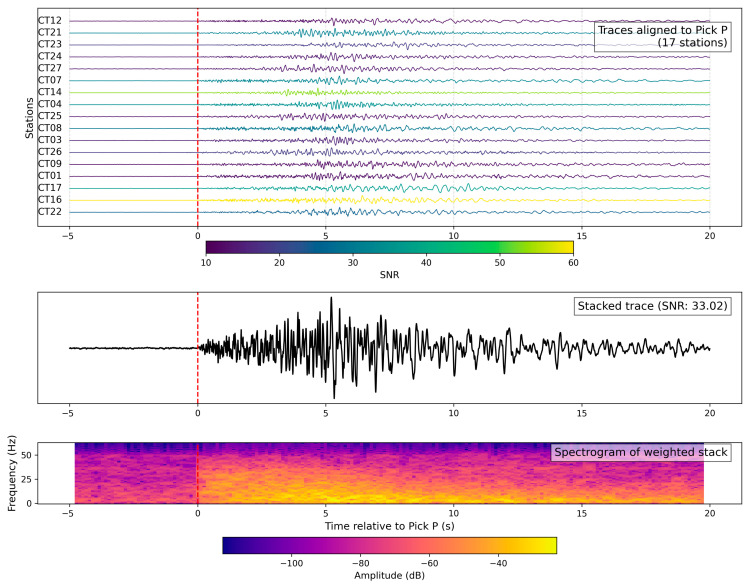
Local event stack (Lioni, 4 April 2025). SNR-weighted stack of vertical-component seismograms recorded by the array for the ML 2.4 earthquake near Lioni (Avellino province) on 4 April 2025 at 18:06:53 UTC. (**Top panel**): individual traces aligned on the manually picked P-wave arrival (red dashed line); color scale reflects each trace’s signal-to-noise ratio (SNR), with low-SNR traces removed (threshold: SNR > 11). (**Middle panel**): weighted stack obtained by averaging normalized traces using their SNR as weights; the resulting stacked trace exhibits an SNR of 34.0. (**Bottom panel**): spectrogram of the stacked trace, showing high-frequency energy (up to 15 Hz) concentrated around the P-wave onset, with a decaying coda at lower frequencies.

As an example, Figure 13 shows the results of the SNR-weighted stack analysis for the ML 2.2 earthquake that occurred on 4 April 2025 at 18:06:53 UTC near Lioni (Avellino province; 40.8387° N, 15.2125° E; depth 15 km). The waveforms from 17 stations were aligned on the P-wave arrival time and stacked using a weight proportional to their signal-to-noise ratio (SNR) without applying any filter. Only traces with SNR > 11 were included. The top panel shows the individual traces aligned on the P pick, color-coded by SNR. The middle panel displays the resulting stacked waveform, with a measured SNR of 34.0. The bottom panel presents the spectrogram of the stacked signal, revealing that the energy is mostly concentrated in the 2–15 Hz range. The high-frequency content associated with the P-wave is evident, followed by a lower-frequency coda. This procedure enhances signal coherence and allows for better characterization of weak local events.

**Figure 14:**
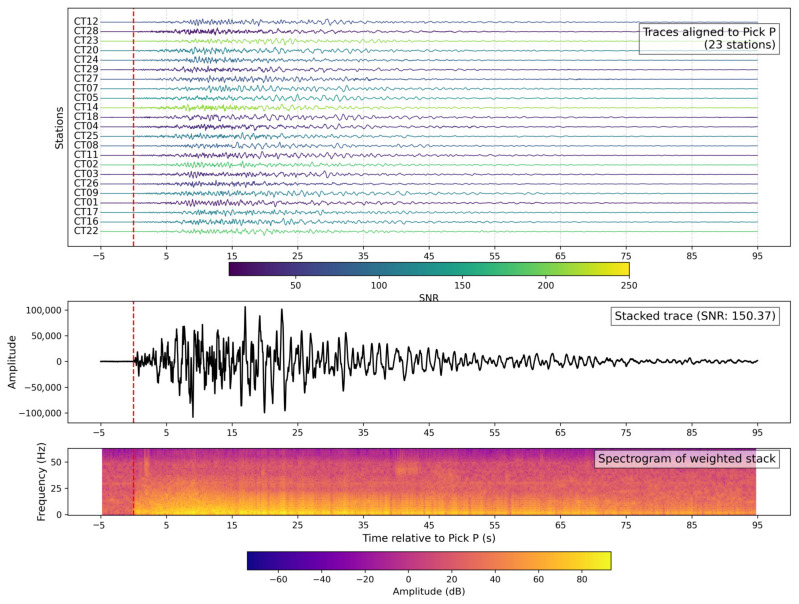
Regional event stack (Potenza, 18 March 2025). SNR-weighted stack of vertical-component seismograms for the Mw 4.2 earthquake in the Potenza province on 18 March 2025 at 09:01:25 UTC. (**Top panel**): aligned traces from 23 stations, colored by SNR; the P-wave onset is used as the reference (red dashed line). (**Middle panel**): weighted stack shows a maximum amplitude of ~3 × 10^5^ counts and an SNR of 163.8, indicating a high-quality, coherent signal. (**Bottom panel**): spectrogram highlights broadband energy up to 25 Hz, with a dominant frequency band below 15 Hz and a prolonged coda lasting over 40 s.

Figure 14 presents an example through the SNR-weighted stack for the Mw 4.2 earthquake that occurred on 18 March 2025 at 09:01:25 UTC in the Potenza province (40.6632° N, 15.8432° E), approximately 50 km east of Contursi Terme. A total of 26 high-quality waveforms were aligned on the P-wave pick and stacked with weights proportional to their SNR. As shown in the top panel, the individual traces exhibit high coherence, with SNR values color-coded and ranging up to ~250. The stacked trace (middle panel) reached a maximum amplitude of ~3 × 10^5^ counts and an overall SNR of 163.8, nearly one order of magnitude higher than in the Lioni example. The spectrogram (bottom panel) displays significant energy up to ~25 Hz, with dominant content below 15 Hz and a coda extending beyond 40 s. This highlights the ability of the array and stacking procedure to enhance the detection and characterization of moderate regional events.

These two case studies highlight the performance of the temporary array in detecting local earthquakes across a range of magnitudes. Even the relatively small Lioni event (ML 2.2) was well resolved above the ambient-noise level, while the Potenza earthquake (Mw 4.2) demonstrates the capability of the network to capture strong motions with high signal-to-noise ratios. This confirms the suitability of the installation for earthquake monitoring, and, more generally, for assessing seismic detectability in the Contursi Terme hydrothermal system.

## 4. Conclusions

In this study, we have presented a seismic array experiment carried out in the area between the municipalities of Oliveto Citra, Colliano, and Contursi Terme, in Southern Italy, where a low-enthalphy hydrotermal system is located, along with the data acquired during the survey. The seismic array deployment was devoted to mainly collect continuous recordings of ambient seismic noise data over a period of about one month in order to mainly pursue two aims of the TOGETHER project that are: (1) to provide a multi-parametric imaging of Contursi Terme subsoil, for an assessment of the geothermal potential of this area; (2) to increase the awareness of citizens about the usage of geothermal energy for tackling the present climatic emergency. Furthermore, from a seismic risk mitigation perspective, which constitutes a collateral element in any potential geothermal project, the continuous acquisition allowed us to: (3) characterize the local seismic response of the different sites onto which the sensors have been installed; (4) test the earthquake detectability of local and regional seismic events by stations located in an urban–rural mixed environment.

Before the deployment of the seismic array, we investigated the potential of an ambient-noise study in the target area. For this purpose, we analyzed the coherency of cross-correlation functions as a function of interstation distances. The cross-correlations were computed on the ambient seismic noise recordings at station pairs belonging to a seismic array of the DETECT experiment, carried out in 2021–2022 along the fault system responsible for the 1980 Irpinia earthquake and located 5 km north-east of the target area. Despite the sensors adopted in the experiment (4.5 Hz geophones), we still retrieved a good coherency of the Green’s function up to about 0.75 km interstation distance, over which the noise becomes dominant on the cross-correlogram.

On these grounds, we proceeded to the installation of twenty-nine 0.5 Hz Sentinel GEO MKII six-component instruments, composed of both accelerometric and velocimetric sensors. We deployed the seismic array trying to meet geometrical criteria that are: (a) interstation distances ranging from 0.5 to about 10 km, distributed as homogeneously as possible; (b) a distribution spanning the whole range of azimuths (0–360°). Furthermore, we covered areas with different surface geological conditions.

The performance of the seismic array was good, both in terms of continuous data availability and characteristics of the background noise. As to the former, indeed, 25 out of 29 seismic stations recorded 100% of data. As to the latter, the computed PPSD display levels of the noise usually between the reference Peterson’s NHNM and NLNM curves; only some noisiest stations exhibit noise amplitudes slightly exceeding the NHNM curve for periods ranging from 0.1 s to 1 s. The spectrogram and the PSD time series, for their part, allowed us to observe the common day–night noise levels oscillations in the highest frequency range (7.2 Hz–14 Hz), whereas some amplitude peaks in the 0.5 Hz–1 Hz curve are consistent with the windiest days that occurred in the acquisition month. Despite the good performance in terms of acquisition continuity and spectral noise signatures, when we dismantled the array, we noticed sensors’ misorientation (with respect to the north) affecting 4 out of 29 stations. Furthermore, in the initial analysis of the data, we observed the malfunctioning of one horizontal component for three sensors of the array, thus extending the problematic stations to 6. In particular, CT06 experienced both an accidental sensor rotation and damage to its longitudinal component. Finally, we observed a small spurious 1 Hz peak on both the longitudinal and vertical components of all the sensors adopted in the experiment; we demonstrated that a proper smoothing procedure of the velocity spectra is the simplest solution to cancel out this peak. Despite these problems, the experiment proved invaluable for testing the sensors’ ability to continuously record seismic data over extended periods and for identifying potential malfunctions. Operating in uncontrolled sites, such as public offices, private homes, and schools (and without dedicated LAN connections), revealed the need to upgrade the instrumentation by adding an internal 4G modem to enable remote control of each station.

Therefore, we initially excluded the stations affected by the above-mentioned problems from our next analyses, which consisted of: (a) estimating the resonance frequencies of the shallow subsoil of the installation sites by the HVSR technique; (b) detecting both local and regional earthquakes that occurred during the experiment. The HVSR study highlighted some regions of the target area with a more prominent amplification effect, mainly in correspondence with terraced eluvial deposits, eluvial/colluvial materials, and clayey units, and particularly in the Bagni di Contursi area, where most of the spa facilities are located.

Finally, the installed seismic array allowed the recording of 35 earthquakes (both local and regional) in the magnitude range 1.4–4.2 over the acquisition period. It testifies to the potential of such an array to monitor and detect seismicity in a context of seismic risk management related to geothermal resource exploitation.

To fully exploit the potential of the recorded dataset, in the near future, we plan to gain insights into the orientation errors for the station affected by this problem. Methodologies like those proposed by [66,67], and successfully applied in [68], which are based on the cross-correlation of seismic signals, will be tested and applied to the collected data. Nevertheless, we are aware of the possible difficulties that we could encounter because of the high cut-off frequency (2 s) of the Sentinel GEO MKII. Concerning the highlighted spurious 1 Hz peak on the longitudinal and vertical components, the spectral whitening procedure suggested by [69] and commonly adopted in the pre-processing of ambient seismic data for tomographic studies (e.g., [43,70,71]) could be a further solution to cancel the effect of this anomaly on the continuous data stream.

Summing up, the continuous seismic data collected through this experiment laid the foundations for a future ambient-noise tomography of the area. It aims at imaging the subsoil in the context of a further assessment of the geothermal potential of the Contursi Terme area. The availability of a future elastic model, combined with ERT, Deep ERT (DERT), and Magnetotelluric models carried out in the framework of the TOGETHER project, will help in constraining the interpretation of subsoil images. This high-quality array dataset also represents a valuable resource for the broader scientific community, enabling a variety of research studies, including earthquake source characterization, seismic hazard assessment, attenuation or anisotropy analyses, as well as the validation of novel array-based seismic processing methodologies.

It is still worth reminding that this experiment could not have been possible without the availability of citizens, public administrations, schools, and private activities of the municipalities involved in the installation of the array. Their active collaboration, which was realized through hosting the seismic sensors and powering them (at their expenses) in their private sites, has been an evident example of a citizen-science action [72]. Moreover, it represents an inspiring chapter in the ongoing story of seismic array development—one that began during the Cold War and now continues in the spirit of synergy and alliance between different actors of civil society.

## Figures and Tables

**Table 1:** Information on the seismic stations installed in the target area.

Station	Latitude	Longitude	Sensor ID	Date of Installation	Date of Removal
CT01	40.64824	15.24495	GE178	5 March 2025	8 April 2025
CT02	40.64948	15.24892	GE183	5 March 2025	8 April 2025
CT03	40.64537	15.23685	GE181	6 March 2025	8 April 2025
CT04	40.65176	15.23152	GE188	7 March 2025	8 April 2025
CT05	40.65991	15.24724	GE193	6 March 2025	8 April 2025
CT06	40.64749	15.26447	GE194	6 March 2025	8 April 2025
CT07	40.64216	15.25548	GE196	6 March 2025	8 April 2025
CT08	40.64091	15.26738	GE185	6 March 2025	8 April 2025
CT09	40.63342	15.26548	GE179	6 March 2025	8 April 2025
CT10	40.63696	15.24393	GE195	6 March 2025	8 April 2025
CT11	40.64460	15.21476	GE184	6 March 2025	4 April 2025
CT12	40.65066	15.21429	GE207	6 March 2025	8 April 2025
CT13	40.66180	15.22233	GE186	3 March 2025	4 April 2025
CT14	40.67775	15.23876	GE192	7 March 2025	8 April 2025
CT15	40.67969	15.2021	GE182	3 March 2025	8 April 2025
CT16	40.70068	15.21637	GE175	6 March 2025	8 April 2025
CT17	40.70182	15.23925	GE176	6 March 2025	8 April 2025
CT18	40.70753	15.25347	GE191	7 March 2025	4 April 2025
CT19	40.71276	15.27786	GE174	6 March 2025	8 April 2025
CT20	40.69769	15.27836	GE203	6 March 2025	8 April 2025
CT21	40.68278	15.30773	GE206	6 March 2025	8 April 2025
CT22	40.67735	15.27076	GE173	6 March 2025	8 April 2025
CT23	40.67281	15.26728	GE204	6 March 2025	8 April 2025
CT24	40.67036	15.24942	GE201	6 March 2025	8 April 2025
CT25	40.68617	15.23277	GE187	6 March 2025	8 April 2025
CT26	40.69054	15.23023	GE180	3 March 2025	8 April 2025
CT27	40.69563	15.25458	GE198	6 March 2025	8 April 2025
CT28	40.68767	15.2515	GE205	6 March 2025	8 April 2025
CT29	40.68291	15.25125	GE199	6 March 2025	8 April 2025

**Table 2:** Local seismic events were detected by the visual inspection of the daily helicorder of the stations composing the seismic array. The event information (i.e., origin time, hypocentral coordinates, magnitude, and location) is extracted from [65].

Origin Time	Latitude Longitude	Depth	Magnitude	Place
4 April 2025 18:06:53	40.8387 15.2125	15	2.4	Lioni (AV)
4 April 2025 18:04:55	40.8463 15.2048	14	1.5	Lioni (AV)
31 March 2025 09:02:42	40.6567 15.8518	11	2.3	Potenza (PZ)
29 March 2025 11:40:27	40.6865 15.7150	11	2.0	Ruoti (PZ)
24 March 2025 02:31:40	40.8113 15.1867	14	2.4	Caposele (AV)
23 March 2025 19:38:07	40.6625 15.8493	11	2.7	Potenza (PZ)
19 March 2025 18:13:16	40.6457 15.8562	10	2.3	Potenza (PZ)
18 March 2025 09:01:25	40.6632 15.8432	13	4.2	Potenza (PZ)
15 March 2025 23:45:14	41.0980 15.2398	7	2.6	Vallesaccarda (AV)
14 March 2025 12:16:37	40.6727 15.8432	14	2.4	Potenza (PZ)
12 March 2025 12:55:22	40.7442 15.4907	9	1.4	Muro Lucano (PZ)

## Data Availability

The seismic array data and the dataless files of the seismic stations are available in the Zenodo repository (https://doi.org/10.5281/zenodo.17343570), subject to a two-year embargo (last accessed on 15 October 2025). The complete seismic dataset belonging to the DETECT experiment and partially adopted in this study is publicly available since 2025-10-01 at https://geofon.gfz.de/doi/network/ZK/2021 (last accessed on 16 October 2025).

## Supplementary Materials

The following supporting information can be downloaded at: https://www.mdpi.com/article/10.3390/s26010016/s1, Figure S1: Probabilistic Power Spectral Densities at all the stations of the seismic array. Figure S2: Time Series of the Power Spectral Densities at all the stations composing the seismic array. Figure S3: Wind speed time series in the target area over the whole period of acquisition. Figure S4: Representation of the 1 Hz spurious peak on the vertical and longitudinal components of the Sentinel GEO MKII.

## Abbreviations

The following abbreviations are used in this manuscript:

GHG	Greenhouse gases
FIR	Finite Impulse Response
RSN	Rete Sismica Nazionale
CC	Cross-correlation
PPSD	Probabilistic Power Spectral Densities
PSD	Power Spectral Densities
NHNM	New-High Noise Model
NLNM	New-Low Noise Model
HVSR	Horizontal-to-vertical spectral ratio
STA	Short Time Average
LTA	Long Time Average
MASW	Multichannel Analysis of Surface
ERT	Electrical Resistivity Tomography
SNR	Signal-to-noise ratio
