# Supplementary material for: Temporary Seismic Array Installation in the Contursi Terme Hydrothermal System: A Step Toward Geothermal Assessment
Source: Sensors (Basel). 2025 Dec 19;26(1):16. doi: 10.3390/s26010016 (PMC12788016; doi:10.3390/s26010016)
Supplement: Supplementary file 1 [file sensors-26-00016-s001.zip › sensors-3960700-supplementary.pdf]

sensors

MDPI

Supplementary material of the manuscript

Temporary Seismic Array Installation in the Contursi Terme Hydrothermal System: A Step Toward Geothermal Assessment

Vincenzo Serlenga [1], Ferdinando Napolitano [2], Serena Panebianco [1], Giovannina Mungiello [2], Tony Alfredo Stabile [1,*], Valeria Giampaolo [1], Massimo Blasone [2], Marianna Balasco [1], Angela Perrone [1], Gregory De Martino [1], Salvatore Lucente [1], Luigi Martino [1,3], Paolo Capuano [2] and Ortensia Amoroso [2]

1 Consiglio Nazionale delle Ricerche, Istituto di Metodologie per l'Analisi Ambientale, 85050 Tito, Italy; vincenzo.serlenga@cnr.it (V.S.); serena.panebianco@cnr.it (S.P.); valeria.giampaolo@cnr.it (V.G.); marianna.balasco@cnr.it (M.B.); angela.perrone@cnr.it (A.P.); gregory.demartino@cnr.it (G.D.M.); salvatorelucente@cnr.it (S.L.); luigimartino@cnr.it (L.M.)
2 Dipartimento di Fisica "E.R. Caianiello", Università degli Studi di Salerno, 84084 Fisciano, Italy; fnapolitano@unisa.it (F.N.); g.mungiello@studenti.unisa.it (G.M.); mblasone@unisa.it (M.B.); pcapuano@unisa.it (P.C.); oamoroso@unisa.it (O.A.)
3 Scuola di Ingegneria, Università degli Studi di Potenza, 85100 Potenza, Italy
* Correspondence: tonyalfredo.stabile@cnr.it

In this document there are reported and briefly commented the figures which are referred to in the main text as Figure S1, Figure S2, Figure S3 and Figure S4.

Figure S1: Probabilistic Power Spectral Densities at all the stations of the seismic array

Figure S2: Time Series of the Power Spectral Densities at all the stations composing the seismic array. The blue and orange curves, which describe the PSD variations in the highest frequency range, highlight the night-day variations at all the stations. This signature is observable at all the components, except for the damaged ones already pointed out in the main text. The amplitude levels differ from a site to another, due to the distinct conditions in the anthropic sources mainly contributing to the level of noise in these frequency ranges. Some stations (e.g. CT01, CT15, CT18, CT26 among the others), which are respectively located in a public school, in a kindergarten, close or inside commercial activities and congested roads, clearly show variations between the work and weekend days, as well.

Figure S3: Wind speed time series in the target area over the whole period of acquisition.

Figure S4: Representation of the 1 Hz spurious peak on the vertical and longitudinal components of the Sentinel GEO MKII and how the Konno – Omachi smoothing (b constant = 40) significantly contributes to cancel it out. The corresponding spectra on the transverse component is also shown for comparison. The choice of the b constant equal to 40 is conservative, as it is the default smoothing parameter which is commonly used in the processing of seismic signals. In that way we demonstrated that even by applying a moderate smoothing, the anomalous behavior of the sensor at that frequency may be completely neglected. The same anomaly and the same effect of the smoothing on the longitudinal component has been observed.

 https://doi.org/10.3390/s26010016

Figure S1 Probabilistic Power Spectral Densities at all the stations of the seismic array. For each row, from the left to the right, there are displayed the PPSD related to the east-west, north-south, vertical component, respectively. In each plot, the grey solid lines represent the NLNM and NHNM curves. The black solid line identifies the 50th percentile of the probabilistic distribution; the lower and upper dashed solid lines represent the 5th and 95th percentile curves, respectively. The lower green and blue bars indicate the available data and the single PSD measurements that go into the histogram, respectively. The colour palette on the right indicates the probability (in percent) that a certain level of noise is present.

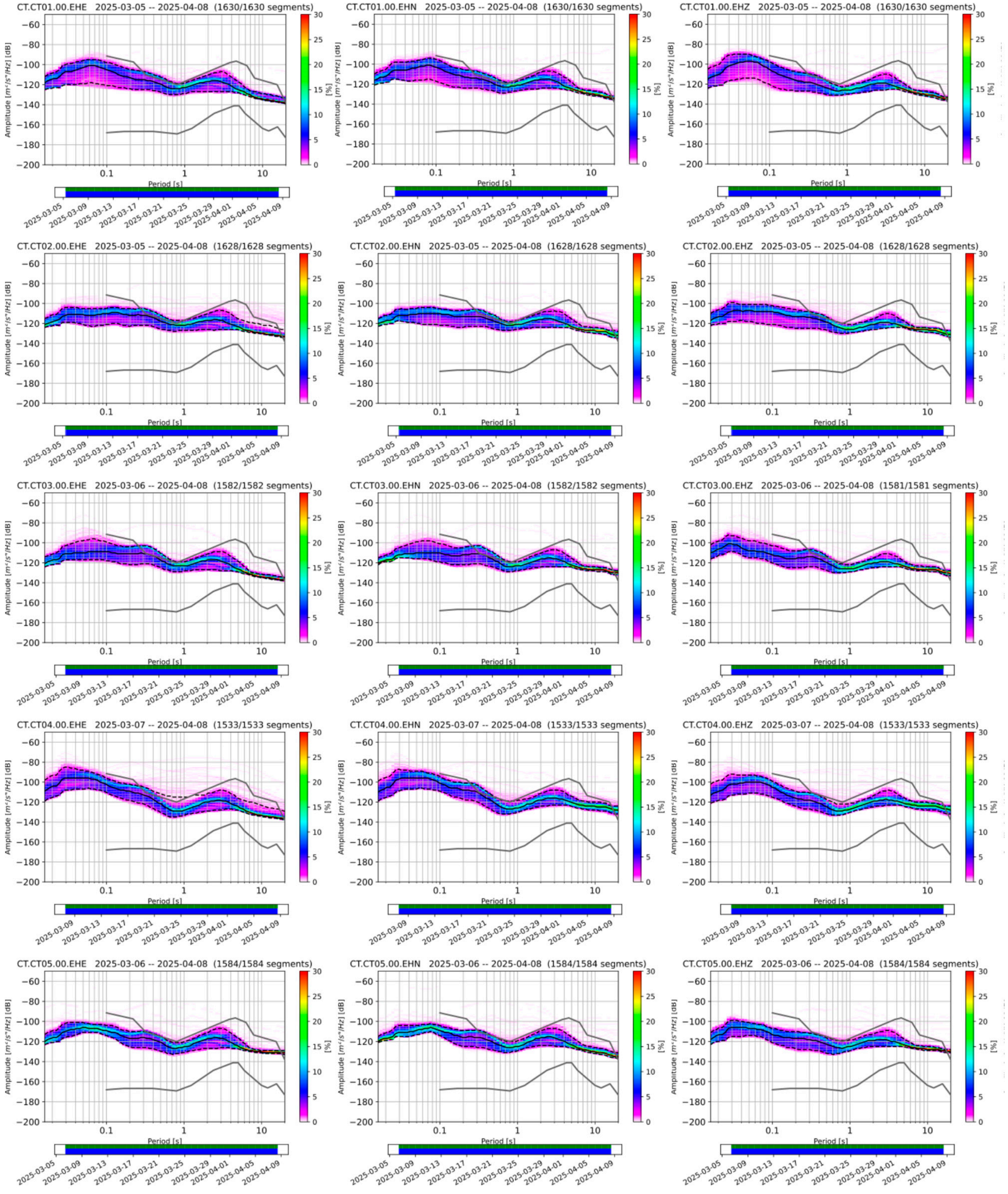

Figure S1a

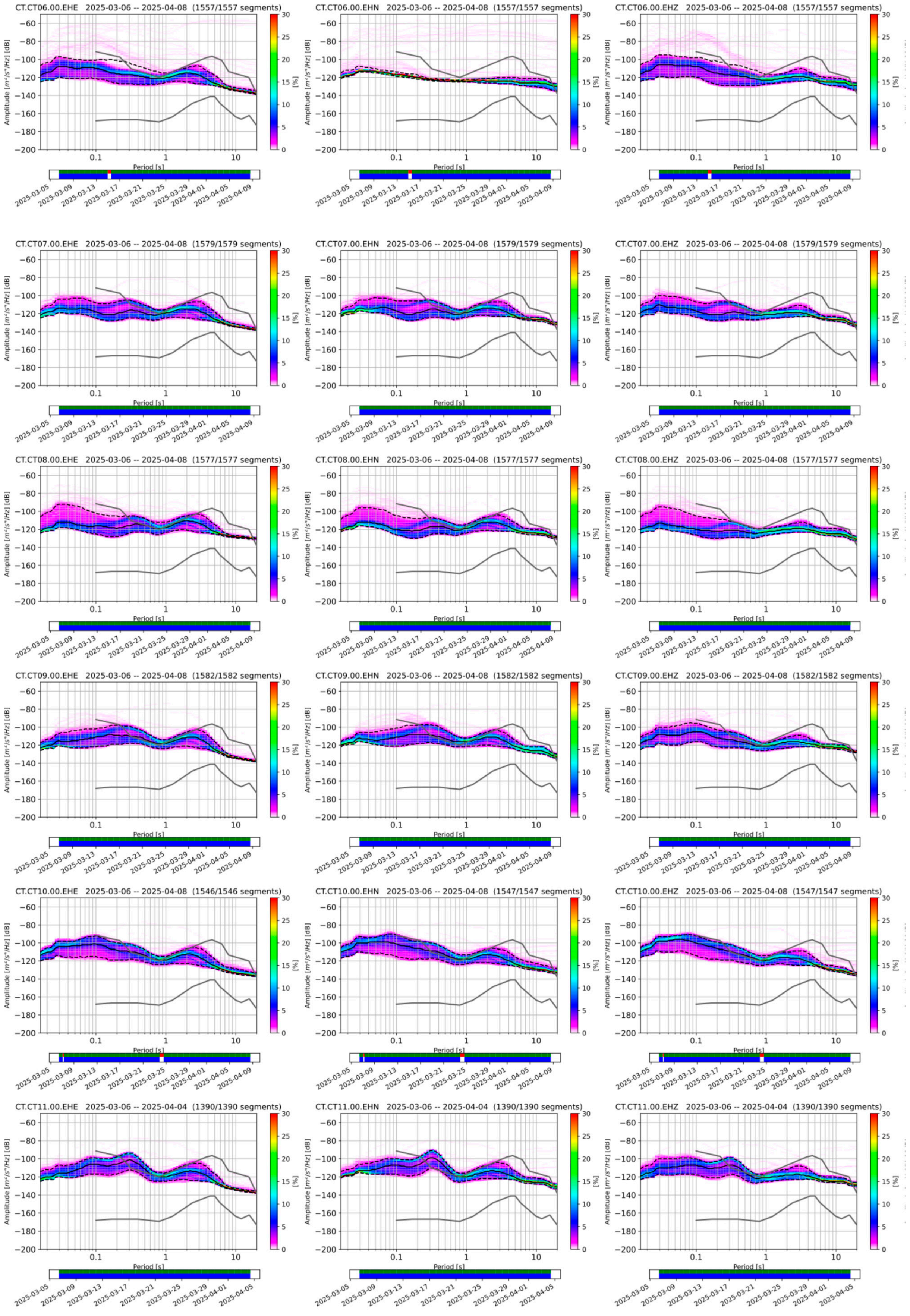

Figure S1b

https://doi.org/10.3390/s26010016

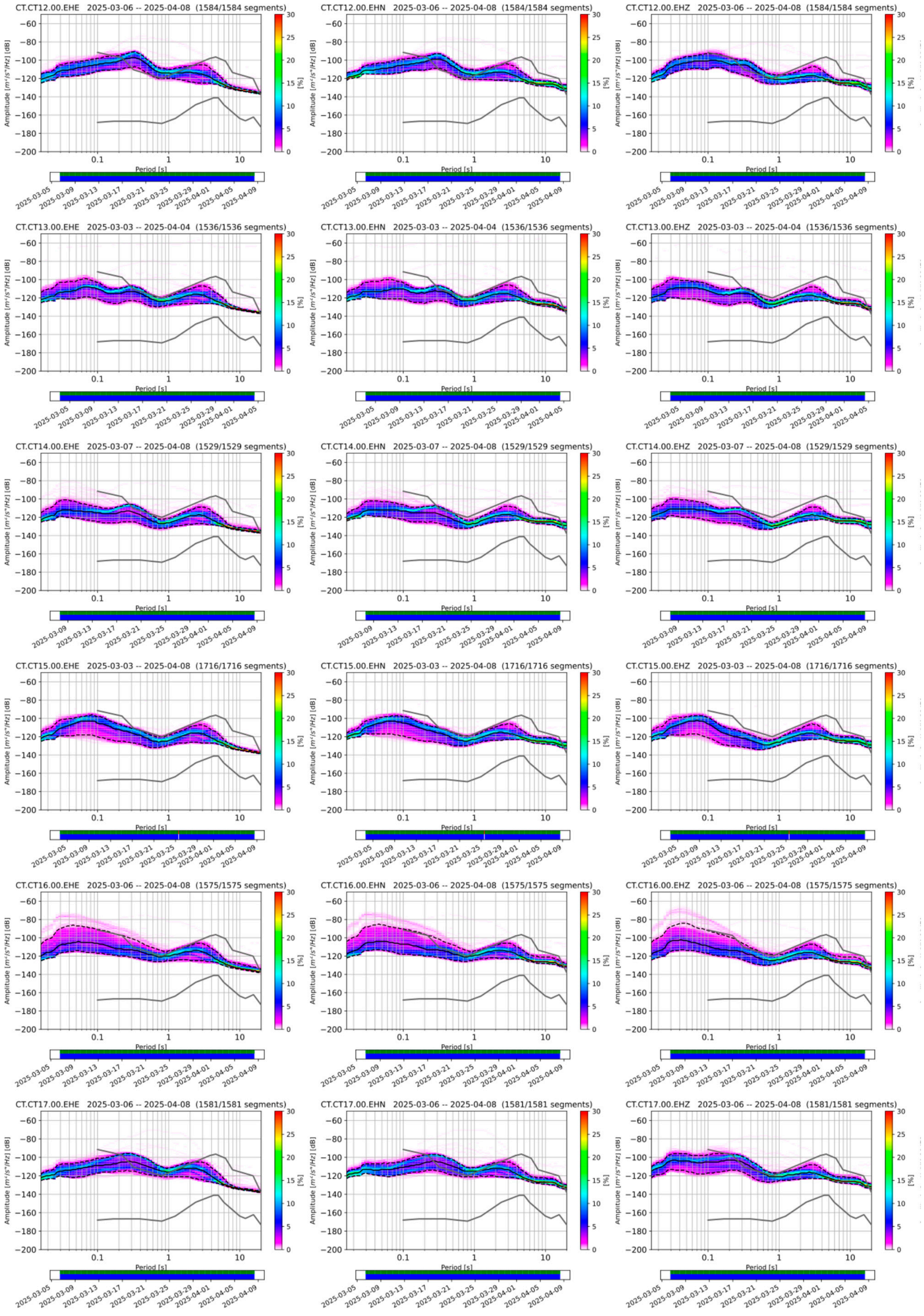
CT.CT12.00.EHE 2025-03-06 -- 2025-04-08 (1584/1584 segments)
CT.CT12.00.EHN 2025-03-06 -- 2025-04-08 (1584/1584 segments)
CT.CT12.00.EHZ 2025-03-06 -- 2025-04-08 (1584/1584 segments)
CT.CT13.00.EHE 2025-03-03 -- 2025-04-04 (1536/1536 segments)
CT.CT13.00.EHN 2025-03-03 -- 2025-04-04 (1536/1536 segments)
CT.CT13.00.EHZ 2025-03-03 -- 2025-04-04 (1536/1536 segments)
CT.CT14.00.EHE 2025-03-07 -- 2025-04-08 (1529/1529 segments)
CT.CT14.00.EHN 2025-03-07 -- 2025-04-08 (1529/1529 segments)
CT.CT14.00.EHZ 2025-03-07 -- 2025-04-08 (1529/1529 segments)
CT.CT15.00.EHE 2025-03-03 -- 2025-04-08 (1716/1716 segments)
CT.CT15.00.EHN 2025-03-03 -- 2025-04-08 (1716/1716 segments)
CT.CT15.00.EHZ 2025-03-03 -- 2025-04-08 (1716/1716 segments)
CT.CT16.00.EHE 2025-03-06 -- 2025-04-08 (1575/1575 segments)
CT.CT16.00.EHN 2025-03-06 -- 2025-04-08 (1575/1575 segments)
CT.CT16.00.EHZ 2025-03-06 -- 2025-04-08 (1575/1575 segments)
CT.CT17.00.EHE 2025-03-06 -- 2025-04-08 (1581/1581 segments)
CT.CT17.00.EHN 2025-03-06 -- 2025-04-08 (1581/1581 segments)
CT.CT17.00.EHZ 2025-03-06 -- 2025-04-08 (1581/1581 segments)
Amplitude [m²/s⁴/Hz] [dB]
Period [s]
[%]

Figure S1c

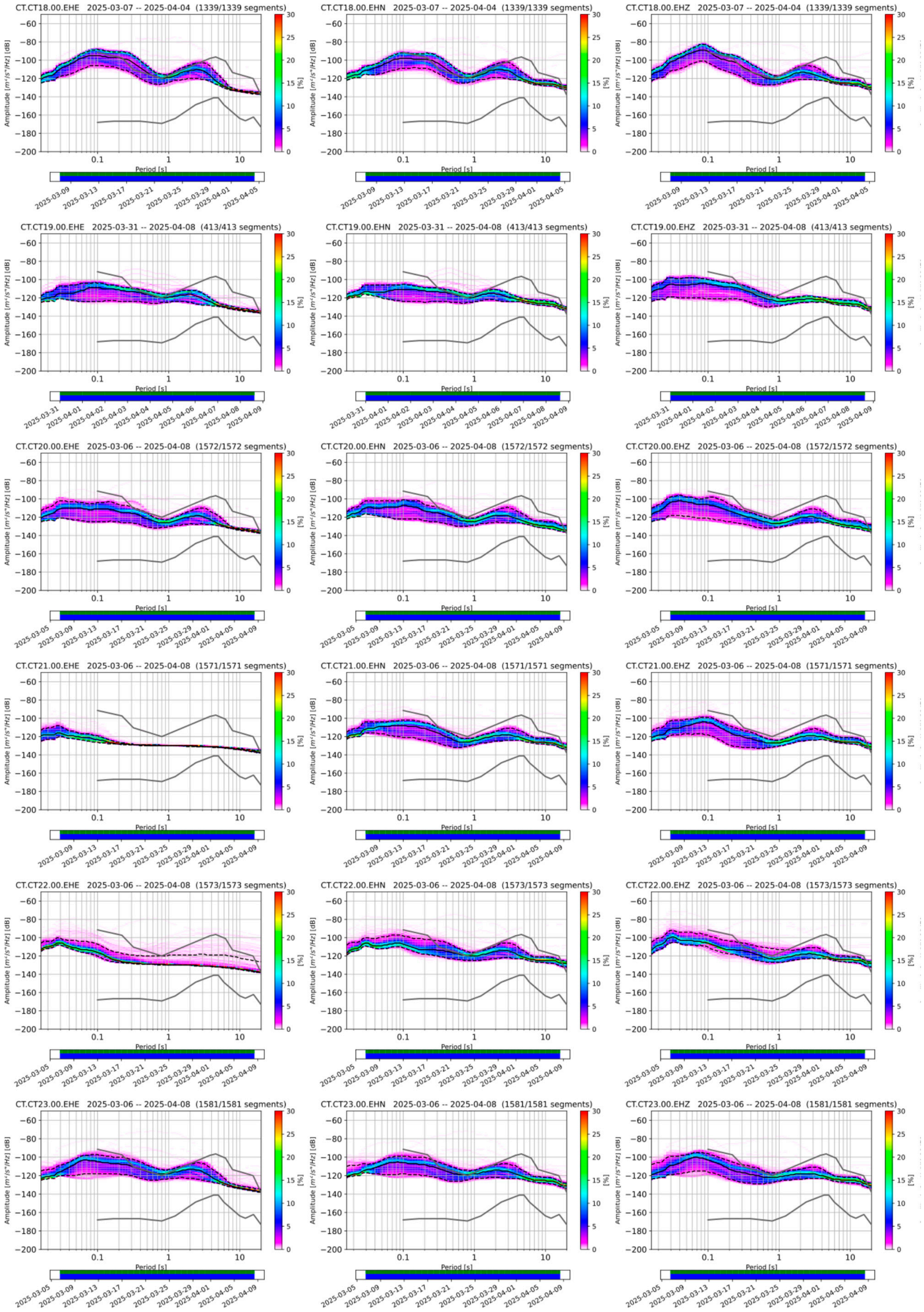
CT.CT18.00.EHE 2025-03-07 -- 2025-04-04 (1339/1339 segments)
CT.CT18.00.EHN 2025-03-07 -- 2025-04-04 (1339/1339 segments)
CT.CT18.00.EHZ 2025-03-07 -- 2025-04-04 (1339/1339 segments)
CT.CT19.00.EHE 2025-03-31 -- 2025-04-08 (413/413 segments)
CT.CT19.00.EHN 2025-03-31 -- 2025-04-08 (413/413 segments)
CT.CT19.00.EHZ 2025-03-31 -- 2025-04-08 (413/413 segments)
CT.CT20.00.EHE 2025-03-06 -- 2025-04-08 (1572/1572 segments)
CT.CT20.00.EHN 2025-03-06 -- 2025-04-08 (1572/1572 segments)
CT.CT20.00.EHZ 2025-03-06 -- 2025-04-08 (1572/1572 segments)
CT.CT21.00.EHE 2025-03-06 -- 2025-04-08 (1571/1571 segments)
CT.CT21.00.EHN 2025-03-06 -- 2025-04-08 (1571/1571 segments)
CT.CT21.00.EHZ 2025-03-06 -- 2025-04-08 (1571/1571 segments)
CT.CT22.00.EHE 2025-03-06 -- 2025-04-08 (1573/1573 segments)
CT.CT22.00.EHN 2025-03-06 -- 2025-04-08 (1573/1573 segments)
CT.CT22.00.EHZ 2025-03-06 -- 2025-04-08 (1573/1573 segments)
CT.CT23.00.EHE 2025-03-06 -- 2025-04-08 (1581/1581 segments)
CT.CT23.00.EHN 2025-03-06 -- 2025-04-08 (1581/1581 segments)
CT.CT23.00.EHZ 2025-03-06 -- 2025-04-08 (1581/1581 segments)
Amplitude [m²/s⁴/Hz] [dB]
Period [s]
[%]

Figure S1d

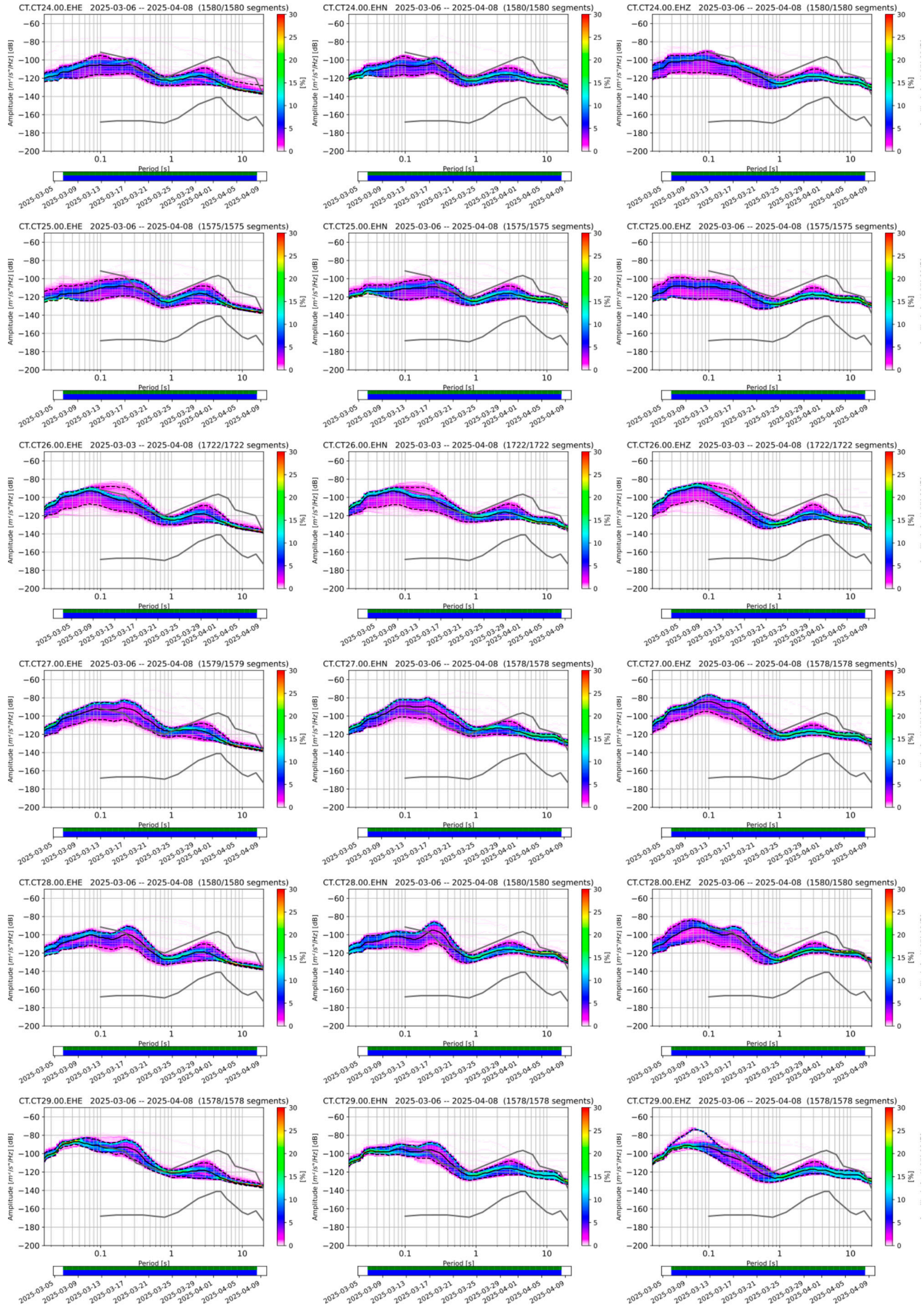
CT.CT24.00.EHE 2025-03-06 -- 2025-04-08 (1580/1580 segments)
CT.CT24.00.EHN 2025-03-06 -- 2025-04-08 (1580/1580 segments)
CT.CT24.00.EHZ 2025-03-06 -- 2025-04-08 (1580/1580 segments)
CT.CT25.00.EHE 2025-03-06 -- 2025-04-08 (1575/1575 segments)
CT.CT25.00.EHN 2025-03-06 -- 2025-04-08 (1575/1575 segments)
CT.CT25.00.EHZ 2025-03-06 -- 2025-04-08 (1575/1575 segments)
CT.CT26.00.EHE 2025-03-03 -- 2025-04-08 (1722/1722 segments)
CT.CT26.00.EHN 2025-03-03 -- 2025-04-08 (1722/1722 segments)
CT.CT26.00.EHZ 2025-03-03 -- 2025-04-08 (1722/1722 segments)
CT.CT27.00.EHE 2025-03-06 -- 2025-04-08 (1579/1579 segments)
CT.CT27.00.EHN 2025-03-06 -- 2025-04-08 (1578/1578 segments)
CT.CT27.00.EHZ 2025-03-06 -- 2025-04-08 (1578/1578 segments)
CT.CT28.00.EHE 2025-03-06 -- 2025-04-08 (1580/1580 segments)
CT.CT28.00.EHN 2025-03-06 -- 2025-04-08 (1580/1580 segments)
CT.CT28.00.EHZ 2025-03-06 -- 2025-04-08 (1580/1580 segments)
CT.CT29.00.EHE 2025-03-06 -- 2025-04-08 (1578/1578 segments)
CT.CT29.00.EHN 2025-03-06 -- 2025-04-08 (1578/1578 segments)
CT.CT29.00.EHZ 2025-03-06 -- 2025-04-08 (1578/1578 segments)
Amplitude [m²/s⁴/Hz] [dB]
Period [s]
[%]

Figure S1e

Figure S2 Time Series of the Power Spectral Densities at all the stations composing the seismic array. For each row, from the right to the left there are reported the PSD relative to the east-west, north-south and vertical component, respectively.

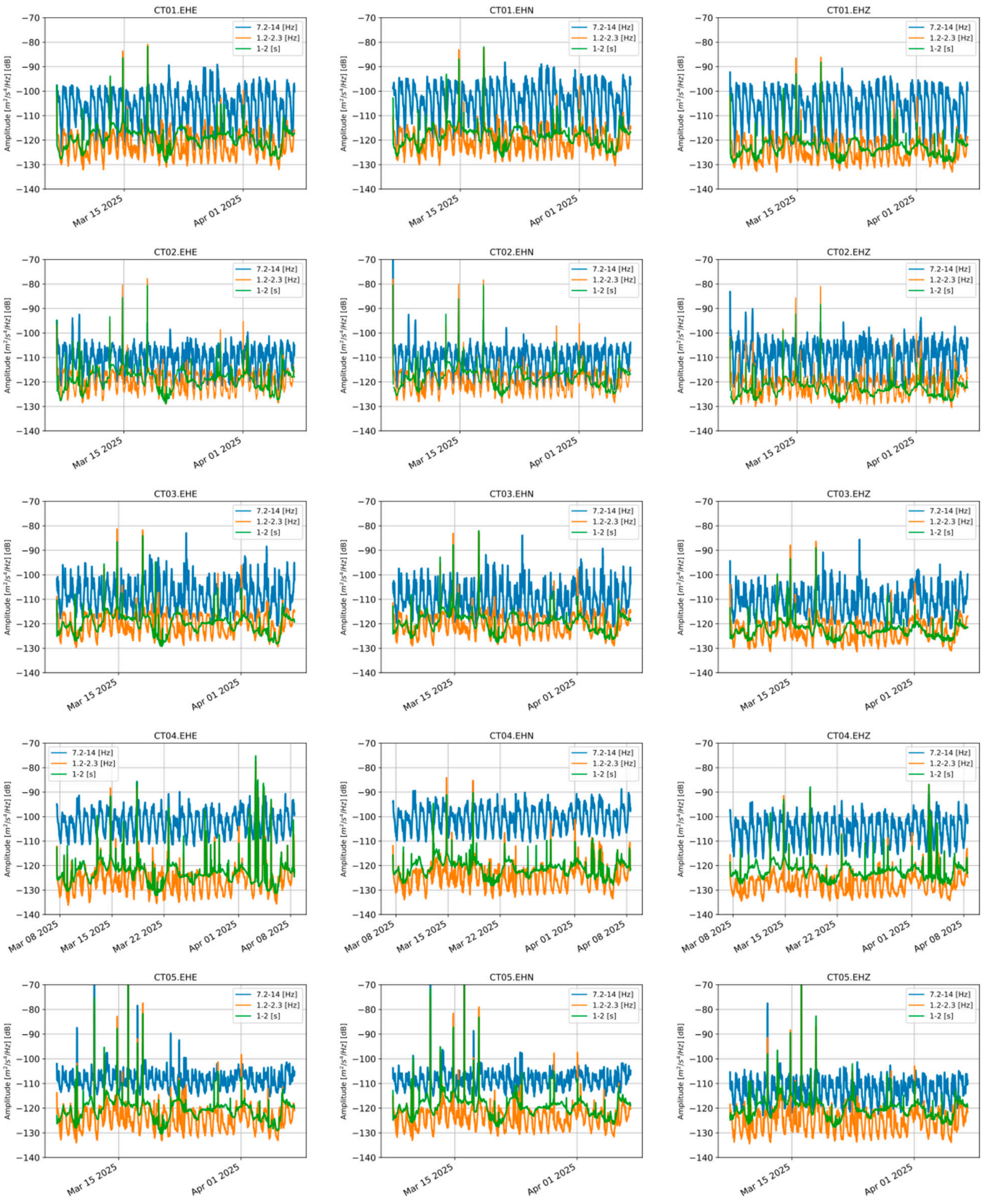

Figure S2a

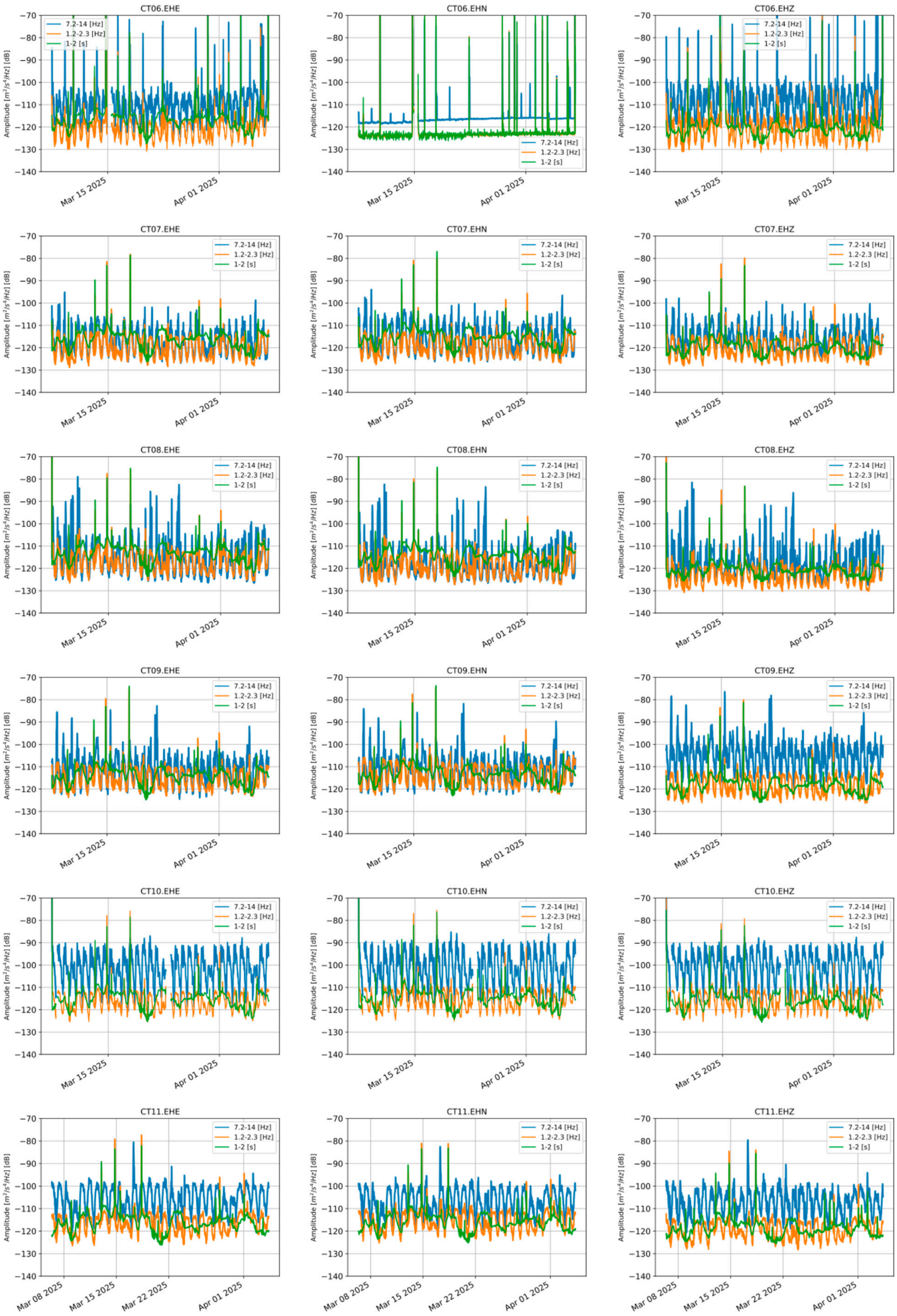
CT06.EHE
CT06.EHN
CT06.EHZ
CT07.EHE
CT07.EHN
CT07.EHZ
CT08.EHE
CT08.EHN
CT08.EHZ
CT09.EHE
CT09.EHN
CT09.EHZ
CT10.EHE
CT10.EHN
CT10.EHZ
CT11.EHE
CT11.EHN
CT11.EHZ
7.2-14 [Hz]
1.2-2.3 [Hz]
1-2 [s]
Amplitude [m²/s⁴/Hz] [dB]
−70
−80
−90
−100
−110
−120
−130
−140
Mar 08 2025
Mar 15 2025
Mar 22 2025
Apr 01 2025

Figure S2b

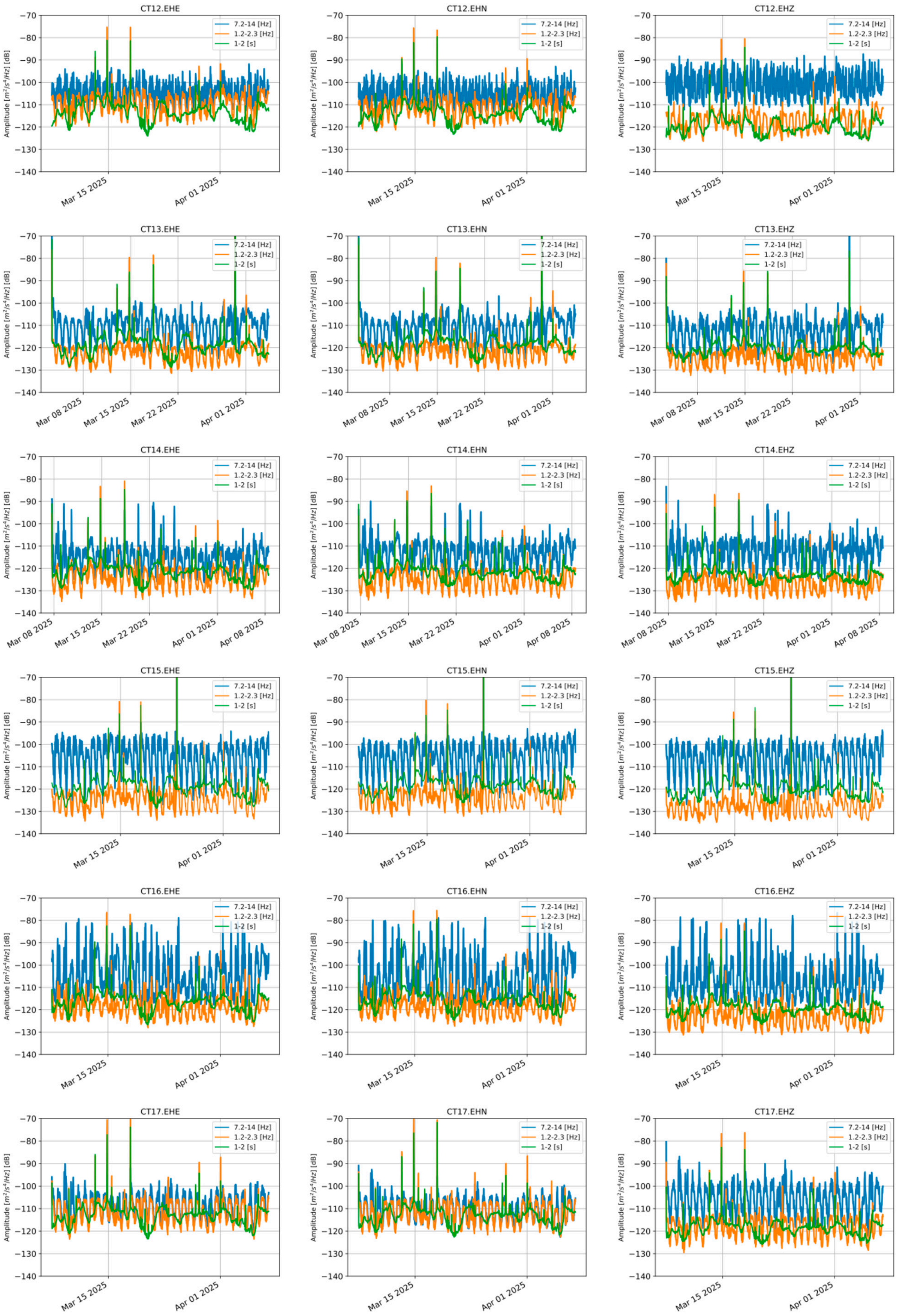
CT12.EHE
CT12.EHN
CT12.EHZ
CT13.EHE
CT13.EHN
CT13.EHZ
CT14.EHE
CT14.EHN
CT14.EHZ
CT15.EHE
CT15.EHN
CT15.EHZ
CT16.EHE
CT16.EHN
CT16.EHZ
CT17.EHE
CT17.EHN
CT17.EHZ
7.2-14 [Hz]
1.2-2.3 [Hz]
1-2 [s]
Amplitude [m²/s⁴/Hz] [dB]
Mar 08 2025
Mar 15 2025
Mar 22 2025
Apr 01 2025
Apr 08 2025

Figure S2c

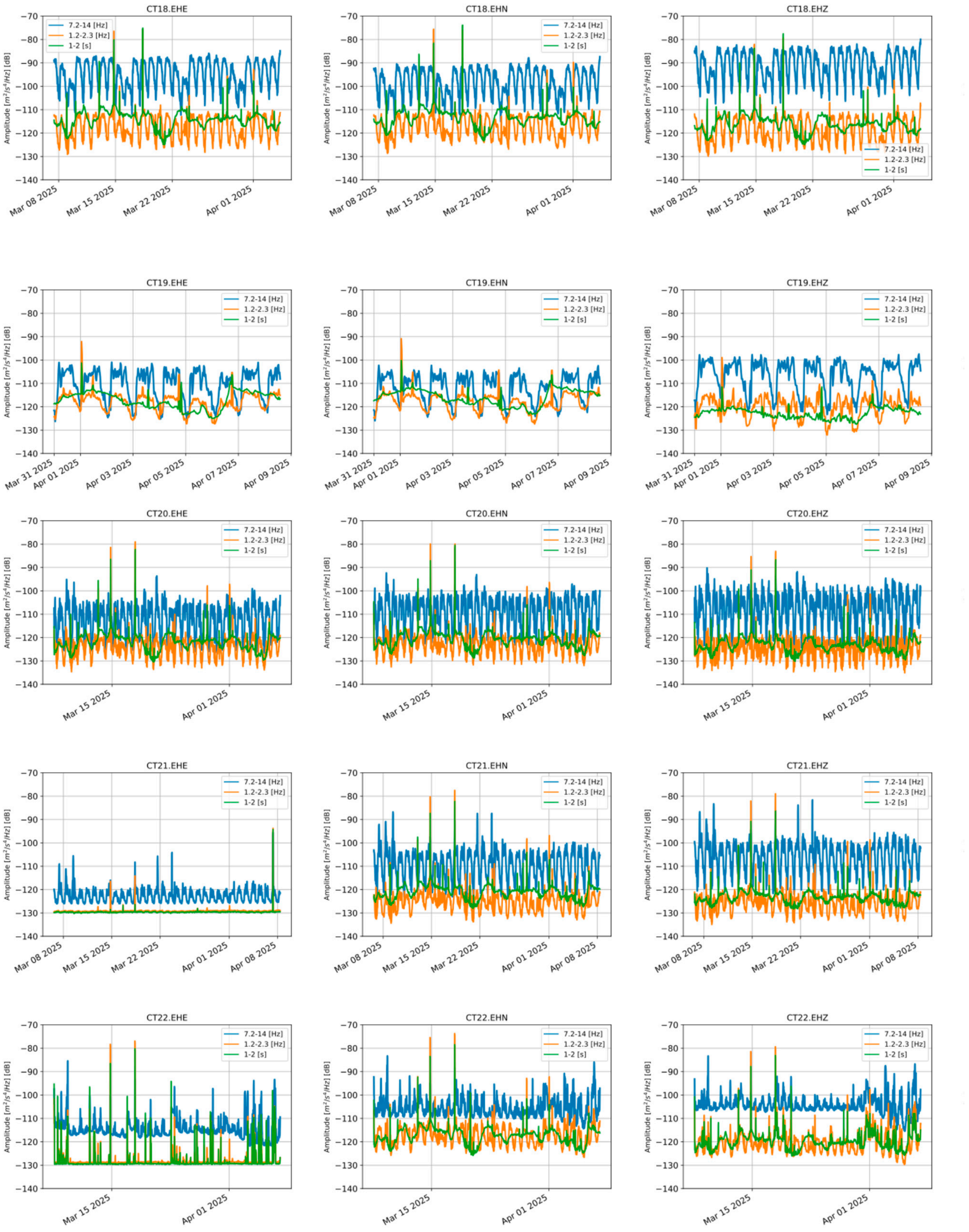
CT18.EHE
CT18.EHN
CT18.EHZ
CT19.EHE
CT19.EHN
CT19.EHZ
CT20.EHE
CT20.EHN
CT20.EHZ
CT21.EHE
CT21.EHN
CT21.EHZ
CT22.EHE
CT22.EHN
CT22.EHZ
7.2-14 [Hz]
1.2-2.3 [Hz]
1-2 [s]
Amplitude [m²/s⁴/Hz] [dB]
−70
−80
−90
−100
−110
−120
−130
−140
Mar 08 2025
Mar 15 2025
Mar 22 2025
Apr 01 2025
Apr 08 2025
Mar 31 2025
Apr 03 2025
Apr 05 2025
Apr 07 2025
Apr 09 2025

Figure S2d

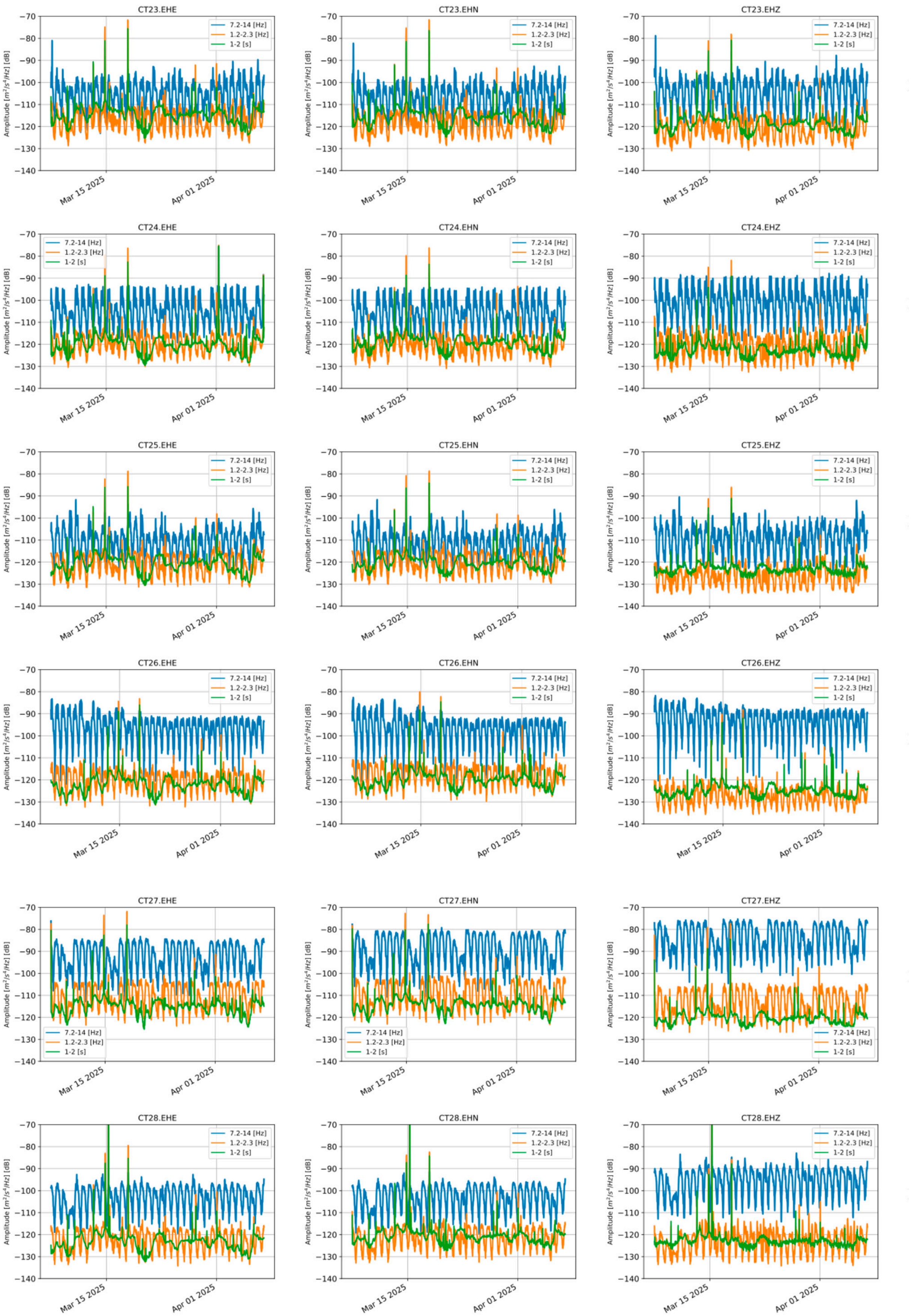
CT23.EHE
CT23.EHN
CT23.EHZ
CT24.EHE
CT24.EHN
CT24.EHZ
CT25.EHE
CT25.EHN
CT25.EHZ
CT26.EHE
CT26.EHN
CT26.EHZ
CT27.EHE
CT27.EHN
CT27.EHZ
CT28.EHE
CT28.EHN
CT28.EHZ
7.2-14 [Hz]
1.2-2.3 [Hz]
1-2 [s]
Amplitude [m²/s⁴/Hz] [dB]
Mar 15 2025
Apr 01 2025

Figure S2d

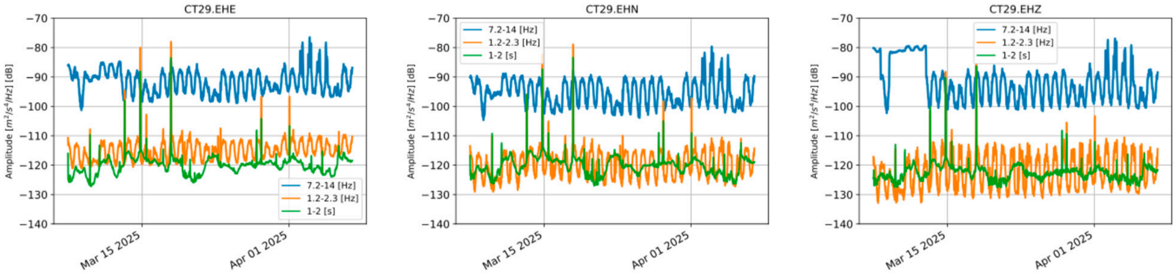

Figure S2e

Figure S3 Wind speed time series in the target area over the whole period of acquisition (1st of March – 8th of April 2025). The data have been extracted in an area between these coordinate extremes: Latmin = 40.63 , Latmax= 40.726856, Longmin=15.327607, Longmax=15.178052.

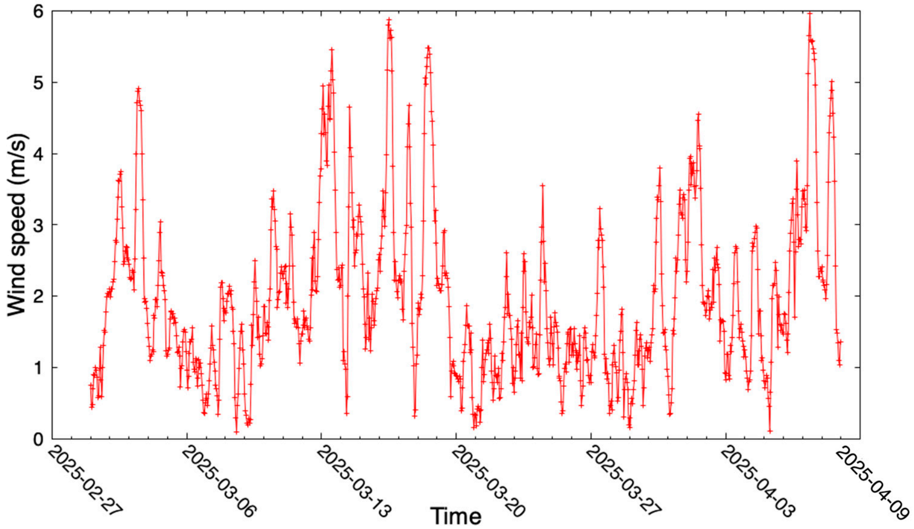

Figure S4 (a) Example of the amplitude spectrum computed on the vertical component (Z) of the velocimetric data recorded between 06:00:00 UTC and 06:17:00 UTC of the 1st of April 2025 at the station CT01 of the seismic array. The purple line describes the raw velocimetric spectrum. The green line depicts the smoothed amplitude spectrum, obtained by adopting the Konno – Ohmachi algorithm, with a value of the b-constant equal to 40 (which is the default value). The black rectangle helps to identify the 1 Hz peak. **(b)** Zoomed representation in the 0.95 Hz – 1.05 Hz frequency range of the amplitude spectra computed on the Z component at all the stations composing the array in the same time interval relative to figure **(a);** the black dashed rectangle aids to identify the anomalous 1 Hz peak, simultaneously observed at all the stations. **(c)** Zoomed representation in the 0.95 Hz – 1.05 Hz frequency range of the smoothed amplitude spectra computed at all the stations composing the array in the same time interval of figure **(a)**. The Konno – Ohmachi algorithm has been adopted with b-constant fixed to 40. **(d)** Example of the amplitude spectrum computed on the longitudinal (North – South) component of the velocimetric data recorded between 06:00:00 UTC and 06:17:00 UTC of the 1st of April 2025 at the station CT01 of the seismic array. The purple line describes the raw velocimetric spectrum. The green line depicts the smoothed amplitude spectrum, obtained by adopting the Konno – Ohmachi algorithm, with a value of the b-constant equal to 40 (which is the default value). The black rectangle helps to identify the 1 Hz peak **(e)** Zoomed representation in the 0.95 Hz – 1.05 Hz frequency range of the amplitude spectra computed on the N-S component at all the stations composing the array in the same time interval relative to figure **(d);** the black dashed rectangle aids to identify the anomalous 1 Hz peak, simultaneously observed at all the stations. **(f)** Zoomed representation in the 0.95 Hz – 1.05 Hz frequency range of the smoothed amplitude spectra computed at all the stations composing the array in the same time interval of figure **(d)**. The Konno – Ohmachi algorithm has been adopted with b-constant fixed to 40. **(g)** Example of the amplitude spectrum computed on the transverse (East - West) component of the velocimetric data recorded between 06:00:00 UTC and 06:17:00 UTC of the 1st of April 2025 at the station CT01 of the seismic array. The purple line describes the raw velocimetric spectrum. The green line depicts the smoothed amplitude spectrum, obtained by adopting the Konno – Ohmachi algorithm, with a value of the b-constant equal to 40 (which is the default value). **(h)** Zoomed representation in the 0.95 Hz – 1.05 Hz frequency range of the amplitude spectra computed on the E-W component at all the stations composing the array in the same time interval relative to figure **(g).**

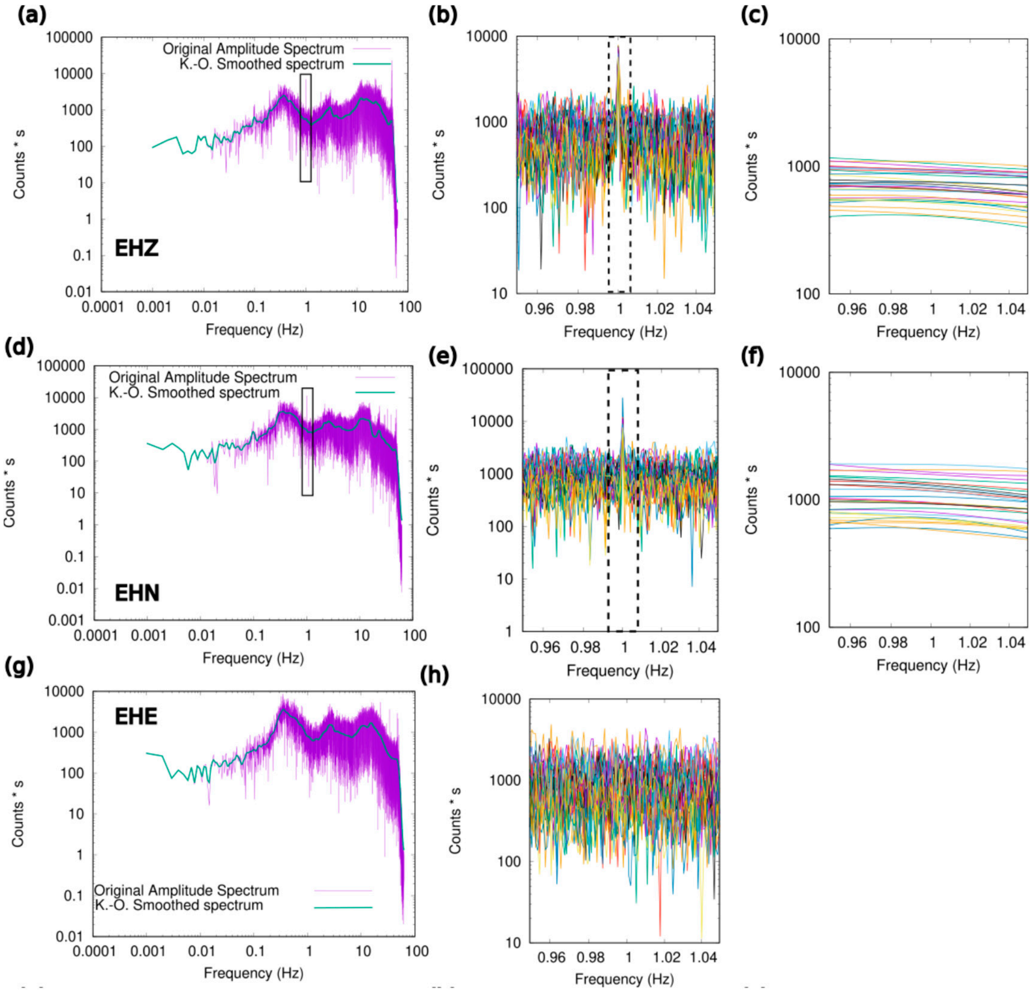

(a)
Original Amplitude Spectrum
K.-O. Smoothed spectrum
EHZ
Counts * s
Frequency (Hz)
(b)
(c)
(d)
EHN
(e)
(f)
(g)
EHE
(h)
